# Psychotic‐Like Experiences in Adolescence Occurring in Combination or Isolation: Associations with Schizophrenia Risk Factors

**DOI:** 10.1176/appi.prcp.20200010

**Published:** 2021-01-18

**Authors:** Alastair G. Cardno, Saskia Selzam, Daniel Freeman, Angelica Ronald

**Affiliations:** ^1^ Division of Psychological and Social Medicine Faculty of Medicine and Health University of Leeds Leeds; ^2^ Social, Genetic and Developmental Psychiatry Centre Institute of Psychiatry, Psychology and Neuroscience King's College London London; ^3^ Department of Psychiatry Oxford Cognitive Approaches to Psychosis (O‐CAP) University of Oxford Oxford Health NHS Foundation Trust Oxford; ^4^ Department of Psychological Sciences Genes Environment Lifespan (GEL) Laboratory Centre for Brain and Cognitive Development Birkbeck, University of London London

## Abstract

**Objectives:**

Individual adolescent psychotic‐like experiences (PLEs) are associated with schizophrenia risk factors. As DSM‐5 schizophrenia requires the co‐occurrence of at least two psychotic symptoms, we investigated whether co‐occurring adolescent PLEs have stronger associations with schizophrenia risk factors, lower quality of life and functioning, and have higher heritability, than individual PLEs.

**Methods:**

Participants were 9646 16‐year‐old twins from the longitudinal Twins Early Development Study. We investigated co‐occurrence of high questionnaire scores for three PLE combinations: (1) paranoia and hallucinations; (2) paranoia or hallucinations, and cognitive disorganization; and (3) paranoia or hallucinations, and negative symptoms, and their associations with 11 schizophrenia‐relevant variables by regression analysis and structural equation twin modeling.

**Results:**

Against expectation, none of the co‐occurring PLEs had the nominally strongest associations significantly more often than individual PLEs. Co‐occurring PLEs had the strongest associations with bullying victimization, cannabis use and lower life satisfaction, but individual PLEs had the strongest associations with cognitive function variables. Obstetric complications were most associated with negative symptoms. Secondary analysis revealed that co‐occurrence of cognitive disorganization and negative symptoms had the nominally strongest associations with most schizophrenia‐relevant variables overall and relatively high heritability (67%).

**Conclusions:**

Focusing on co‐occurrence enhances some individual PLE associations but obscures others. The combination of subjective cognitive disorganization plus observed negative symptoms showed a broad range of enhanced associations with schizophrenia‐relevant variables. Future research could investigate associations with other risk factors and the ability of this PLE combination to predict onset of schizophrenia.

## HIGHLIGHTS

- Focusing on co‐occurring psychotic‐like experiences (PLEs) in adolescence enhances some individual PLE associations but may obscure others
- While victimization, cannabis use, and lower life satisfaction have broad associations with PLE combinations, cognitive variables are most associated with cognitive disorganization and negative symptoms, and obstetric complications are most associated with negative symptoms
- The combination of subjective cognitive disorganization and observed negative symptoms has a relatively broad range of enhanced associations with schizophrenia‐relevant variables and relatively high heritability

There is considerable interest in the relationships between psychotic‐like experiences (PLEs) in the general population and schizophrenia (1, 2), particularly PLEs occurring during adolescence, just prior to the main period of onset of schizophrenia (3, 4, 5). As these PLEs are unaffected by the consequences of having a clinical disorder, such as the effects of antipsychotic medication, they may give new insights into the etiology and conceptualization of schizophrenia, and potentially inform intervention and prevention approaches.

The term PLE covers a wide range of phenomena occurring in the general population, from mild feelings of suspiciousness or unusual perceptions to positive psychotic symptoms such as delusions and hallucinations (3, 6). Broad definitions also include cognitive disorganization and negative symptoms (3). PLEs are also known as psychotic experiences or psychotic experiences and negative symptoms (7), and have some similarities to the concept of schizotypy (8).

Individual PLEs are associated with a range of schizophrenia risk factors (9, 10, 11, 12, 13, 14). DSM‐5 schizophrenia requires the co‐occurrence of at least two psychotic symptoms. Additionally, schizophrenia is associated with lower quality of life and functioning (15, 16), and has higher twin heritability (∼80%) (17, 18) than individual general population PLEs (15%–59%) (14) as well as higher single nucleotide polymorphism heritability (7, 19). This raises the question of whether co‐occurring PLEs have stronger associations with schizophrenia‐relevant variables than their individual component PLEs.

Previous studies have found that, compared to one form of PLE occurring alone, co‐occurrence of delusional and hallucinatory PLEs is associated with poorer functioning (20) and greater risk of psychotic disorder (21, 22), family history of psychosis (23), anxiety symptoms, negative symptoms, childhood trauma, persistence of PLEs, and clinical need (24). Co‐occurrence of positive PLEs and negative symptoms is associated with poorer functioning (25) and greater risk of schizophrenia (2).

We aimed to investigate, for the first time specifically in adolescents, and broadening the range of prospectively assessed risk factors compared to previous studies, whether co‐occurring PLEs have stronger associations with (a) schizophrenia risk factors, (b) lower quality of life and functioning, and (c) have higher twin heritability than individual PLEs.

## Methods

### Participants

Participants were members of the Longitudinal Experiences And Perceptions (LEAP) study (3), that is part of the Twins Early Development Study (TEDS), a general population sample of monozygotic (MZ) and dizygotic (DZ) twins born in England and Wales in 1994–1996 and assessed longitudinally across childhood and adolescence (26). TEDS and LEAP have full ethical approval, and written informed consent was obtained after the procedures had been fully explained at each point of contact.

In total 10,874 families from TEDS were invited to take part in LEAP. Parent reports for 5076 (46.7%) families and twin reports for 5059 (46.5%) pairs were obtained (3). Adolescents involved in the LEAP project had a mean age of 16.3 years (SD 0.68) (3). Individuals were excluded if they did not provide consent at first contact (when TEDS was started) or for this study, had severe perinatal complications, or a severe medical disorder. After exclusions, the sample comprised 9646 individual twins with data on multiple PLEs.

### Psychotic‐Like Experiences

PLEs were assessed using the Specific Psychotic Experiences Questionnaire (SPEQ) at age 16 years (3). SPEQ includes self‐report subscales for paranoia, hallucinations, and cognitive disorganization, and a parent‐rated subscale for negative symptoms. Each subscale includes multiple items relating to the relevant concept (see supplementary methods for further information).

The primary analysis focused on the following PLE combinations: (1) paranoia and hallucinations; (2) paranoia or hallucinations, and cognitive disorganization; and (3) paranoia or hallucinations, and negative symptoms.

We defined PLEs as binary variables, being counted as present if their score was over the threshold closest to the top 15%, and co‐occurring phenotypes as over this threshold for multiple PLEs, for example, for both paranoia and hallucinations. This gave at least 500 individual twins in each PLE group, and in previous analysis of this sample the heritabilities at this threshold were not significantly different from those at more extreme thresholds (14).

### Schizophrenia Risk Factors

Schizophrenia risk factors were as follows: family history (27) measured as presence/absence of schizophrenia in a first or second degree relative (14) and validated with the schizophrenia polygenic risk score; older paternal age (28); ethnic minority status (29); obstetric complications (30); slower developmental milestones (31) measured with total vocabulary at age 2 years; lower premorbid IQ (32) measured as general cognitive ability (g) at age 12 years; bullying victimization (33) at age 12 years (12); and cannabis use (34) by age 16 years (13). Further information is presented in Table 1 and supplementary methods.

**TABLE 1:** Schizophrenia‐relevant variables in the study

	Study variable	How measured^a^
Schizophrenia risk factor		
Family history of schizophrenia	Family history of schizophrenia	Presence/absence of schizophrenia in a first or second degree relative
Older paternal age	Paternal age	Father's age in years when twins born
Ethnic minority status	Ethnicity	White/Other ethnicity
Obstetric complications	Obstetric complications	Composite score of prenatal and neonatal problems
Slower developmental milestones	Vocabulary	Total vocabulary at age 2 years
Lower premorbid IQ	General cognitive ability	Standardized general cognitive ability score (g) at age 12 years
Childhood trauma	Bullying victimization	Total score on the Multidimensional Peer Victimization Scale at age 12 years
Cannabis use	Cannabis use	Whether ever used cannabis by age 16 years
Quality of life and functioning		
Reduced quality of life	Life satisfaction	Overall mean score on the Brief Multidimensional Students' Life Satisfaction Scale
Reduced functioning	GCSE score	Total GCSE points score at age 16 years
Heritability	Twin heritability	Heritability based on an ACE model, comprising additive genetic, common environmental and individual‐specific environmental effects, in monozygotic and same‐sex dizygotic pairs

Abbreviation: GCSE, General Certificate of Secondary Education.

^a^ Further details in supplementary methods.

### Quality of Life and Functioning Variables

Quality of life and functioning variables were life satisfaction measured at age 16 years and General Certificate of Secondary Education (GCSE) attainment at age 16 years, respectively. (See Table 1 and supplementary methods.).

### Analysis

For schizophrenia risk factors, we used logistic regression analysis with the PLE groups as dependent variables. For example, for analysis of paranoia (P) and hallucinations (H) there were four exclusive groups: twins who scored high on neither P nor H (baseline group); those who scored high on P only; those who scored high on H only; and those who scored high on both P and H (P+H). The neither P nor H baseline group was compared with the P only group, the H only group, and the P+H group. The risk factor was the independent variable (with adjustment for birth order, age, and sex). We also adjusted for socioeconomic status at first contact whenever there were significant associations. We included all twins and adjusted for clustering within twin pairs using a generalized estimating equations approach.

We hypothesized that the co‐occurring PLE group (e.g., P+H) would have the at‐least nominally highest odds ratio (OR) for each risk factor. Where this was the case, we conducted a post hoc logistic regression analysis between the co‐occurring group (e.g., P+H) and the group with the next highest odds ratio (e.g., P only) to determine if the association for the co‐occurring group was significantly greater than for the group with the next strongest association.

For analyses of life satisfaction and reduced functioning, we used linear regression analysis with the life satisfaction or GCSE score as the dependent variable, and the PLE groups as the independent variable.

For initial description of the results, statistical significance was taken as p<0.05, two‐tailed, with thresholds of p<0.01 and p<0.001 also reported.

### Heritability Estimates

These were based on MZ and same‐sex (SS) DZ twin pairs, following the approach used previously in TEDS (14), and most twin studies of schizophrenia (17, 18). Zygosity of twins was determined either using parental questionnaires (accuracy >95%) or DNA (26).

The twin design involves comparing within‐pair similarities of MZ and DZ twin pairs to determine the extent that variation in a phenotype is attributable to genetic and environmental influences. For a detailed explanation of the twin model please see (35). We calculated probandwise concordances and tetrachoric correlations for each PLE group, for example, P only, H only, and P+H, in MZ and SS DZ pairs, to obtain an initial impression of the MZ and DZ similarities. We then carried out univariate model‐fitting of the PLE groups based on a liability‐threshold ACE model to establish the relative contribution of additive genetic (A), common environmental (C) and individual‐specific environmental influences (E) (14). We treated heritability (h^2^) estimates as statistically significant at p<0.05 if their 95% confidence intervals (95% CI) did not include zero, and two h^2^ estimates as significantly different if their 95% CIs did not overlap.

### Overall Test of Associations with Schizophrenia‐Relevant Variables

The primary study outcome was whether the co‐occurring PLE group (e.g., P+H) had the at‐least nominally strongest association with significantly more of the 11 schizophrenia‐relevant variables analyzed than its individual component PLEs (e.g., P only, or H only), where at least one PLE group was significantly associated. A one‐sample binomial test was used compared with a null proportion of 1 in 3 (0.33; e.g., in a comparison of P+H, P only and H only, the P+H group would be expected to have the strongest association 1 in 3 times by chance).

The threshold for statistical significance was p<0.017, two‐tailed, for these overall tests (p<0.05 with Bonferroni adjustment for three sets of analyses (1) paranoia and hallucinations; (2) paranoia or hallucinations, and cognitive disorganization; and (3) paranoia or hallucinations, and negative symptoms).

### Analysis Software

We used SPSS version 24 (https://www.ibm.com/products/spss-statistics) for the analysis of risk factors, quality of life and functioning variables, and calculation of probandwise concordances. We used OpenMx (https://openmx.ssri.psu.edu/) for calculating tetrachoric correlations and twin modeling analyses.

## Results

Descriptive statistics are shown in Table 2. Paranoia, hallucinations, and cognitive disorganization were more common in females, while negative symptoms without paranoia or hallucinations was more common in males, consistent with previous reports (3). Family socioeconomic status at first contact was lower in individuals with negative symptoms, and to a lesser extent also in individuals with cognitive disorganization, and hallucinations (All p‐values<0.001: Table 2).

**TABLE 2:** Demographic variables for PLE groups

	Demographic variable						
	No. individual twins		Sex^b^	Age in years		SES at first contact^c^	
PLE Group^a^	*N*	%	% Male	Mean	SD	Mean	SD
Paranoia (P) and hallucinations (H) (*N* = 9646)							
Neither P nor H	7217	74.8	45.6	16.3	0.68	0.25	0.99
P and not H	814	8.4	42.1	16.4	0.67	0.22	1.02
H and not P	986	10.2	41.2	16.3	0.67	0.11	0.96
Both P and H	629	6.5	38.5	16.3	0.72	0.15	1.00
Cognitive disorganization (CD) and paranoia or hallucinations (P/H) (*N* = 9629)							
Neither CD nor P/H	6633	68.9	46.9	16.3	0.68	0.27	0.98
CD and not P/H	572	5.9	31.1	16.3	0.68	0.11	0.99
P/H and not CD	1672	17.4	45.3	16.3	0.68	0.20	0.99
Both CD and P/H	752	7.8	30.7	16.3	0.68	0.07	0.99
Negative symptoms (NS) and paranoia or hallucinations (P/H) (*N* = 9579)							
Neither NS nor P/H	6147	64.2	44.3	16.3	0.68	0.29	0.98
NS and not P/H	1018	10.6	53.7	16.3	0.68	0.03	0.98
P/H and not NS	1844	19.3	40.7	16.3	0.67	0.25	0.98
Both NS and P/H	570	6.0	41.1	16.3	0.70	‐0.13	0.98

Abbreviations: PLE, psychotic‐like experience; SES, family socioeconomic status as a standardized composite score based on parental qualifications and employment ‐ higher score means higher socioeconomic status.

^a^ PLE was defined as present if scoring in the top ∼15% of the sample.

^b^ Sex distributions differed significantly for all three PLE groupings: P and H (χ^2^ = 19.20, df 3, p<0.001); CD and P/H (χ^2^ 115.29, df 3, p<0.001); NS and P/H (χ^2^ = 48.59, df 3, p<0.001).

^c^ SES distributions differed significantly for all three PLE groupings: P and H (F = 6.93, df 9136, p<0.001); CD and P/H (F = 12.68, df 9121, p<0.001); NS and P/H (F 42.76, df 9074, p<0.001).

### Primary Analysis

Results are summarized in Table 3 and detailed in Tables [Link], [Link], [Link] of the online supplement. In none of the three primary analyses did the co‐occurring PLE group have the strongest association with significantly more variables than its individual component PLEs overall (last row of Table 3: p‐values 0.16–0.39).

**TABLE 3:** Primary analysis of associations between PLE groups and schizophrenia‐relevant variables

Schizophrenia‐relevant variable	PLE Groups^a^		
	Paranoia (P) and hallucinations (H)	Cognitive disorganization (CD) and paranoia or hallucinations (P/H)	Negative symptoms (NS) and paranoia or hallucinations (P/H)
Logistic regression analysis (effect measure: odds ratio; no effect = 1)			
Family history of schizophrenia	P+H > P > [H]	CD+P/H* > CD > P/H	NS+P/H* > NS > P/H
	1.46 1.36 0.84	1.72 1.49 1.00	1.78 1.55 1.10
Paternal age	P > P+H > [H]	P/H > [CD] > [CD+P/H]	P/H > [NS] > [NS+P/H]
	1.003 1.000 0.996	1.001 0.999 0.996	1.000 0.992 0.990
Ethnicity	H > P+H > [P]	P/H* > CD > [CD+P/H]	NS** > P/H > NS+P/H
	1.27 1.23 0.95	1.29 1.22 0.75	1.66 1.21 1.13
Obstetric complications	P+H > P > [H]	CD+P/H > [P/H] > [CD]	NS+P/H* > NS** > P/H
	1.79 1.15 0.83	1.57 0.92 0.53	2.38 2.05 1.09
Vocabulary	H* > P+H > [P]	CD** > CD+P/H > P/H	NS* > NS+P/H > P/H
	1.004 1.000 0.997	1.008 1.004 1.001	1.005 1.002 1.001
General cognitive ability	H > [P+H] > [P]	CD*** > CD+P/H** > [P/H]	NS*** > NS+P/H** > P/H
	1.09 0.98 0.95	1.34 1.18 0.98	1.41 1.23 1.01
Bullying victimization	P+H*** >^‡^P***> H***	CD+P/H*** >^‡‡‡^P/H***> CD***	NS+P/H*** >^‡^P/H***> NS**
	1.59 1.42 1.24	1.58 1.35 1.26	1.51 1.37 1.11
Cannabis use	P+H*** > H*** > P***	CD+P/H*** >^‡‡^P/H*** > CD***	NS+P/H*** >^‡‡^P/H*** > NS
	2.61 2.04 1.89	3.15 1.95 1.90	3.10 1.90 1.10
Linear regression analysis (effect measure: β; no effect = 0)			
Life satisfaction	P+H*** >^‡^P*** > H***	CD+P/H*** >^‡‡‡^ CD***> P/H***	NS+P/H*** >^‡‡‡^P/H***>NS***
	0.21 0.19 0.10	0.25 0.15 0.13	0.23 0.15 0.09
GCSE score	H** > P+H** > P	CD*** > CD+P/H*** > P/H	NS*** > NS+P/H*** > P/H*
	2.88 2.86 0.06	8.61 7.33 0.50	10.45 10.16 1.37
Twin heritability (effect measure: h^2^; no effect = 0, maximum = 1)	P^□^ > H > P+H	CD+P/H^□^ > P/H > [CD]	NS^□^ > P/H^□^ > NS+P/H^□^
	0.45 0.21 0.17	0.52 0.24 0.00	0.46 0.33 0.30
Proportion of comparisons where co‐occurring PLE group had strongest association^b^	Proportion: 3/6 (0.50)	Proportion: 5/9 (0.56)	Proportion: 5/10 (0.50)
	p‐value: 0.39	p‐value: 0.16	p‐value: 0.26

*Notes*: > indicates relative sizes of trends. [ ] indicates trend in opposite direction to that expected. *p<0.05, **p<0.01, ***p<0.001 for association with the particular PLE group versus baseline of neither PLE present. Where the group of co‐occurring PLEs (e.g., P+H) had the nominally strongest association, post hoc ^‡^p<0.05, ^‡‡^p<0.01, ^‡‡‡^p<0.001 indicates whether the co‐occurring PLE group had a significantly stronger association than the individual PLE with the next strongest association.

Generalized estimating equations (GEE) logistic or linear regression analysis adjusted for birth order, sex, age ∼16 years when PLE questionnaires completed. Vocabulary age 2 years also adjusted for age ∼2 years when vocabulary assessed. Where significant association additional adjustment for socioeconomic status at first contact.

Heritability (h^2^) based on ACE model, comprising additive genetic, common environmental and individual‐specific environmental effects, in monozygotic and same‐sex dizygotic pairs; □ indicates 95% CIs not including zero and treated as p<0.05.

Abbreviations: GCSE, General Certificate of Secondary Education; PLE, psychotic‐like experience.

^a^ Effects given to two decimal places, expanding to three decimal places where required to show order of effects, and presented such that higher values indicate stronger association with schizophrenia‐relevant variable in the direction found for schizophrenia.

^b^ Proportion of comparisons where the co‐occurring PLE group had the nominally strongest association among the comparisons where at least one association was nominally significant; p‐value is for one sample binomial test, two‐tailed, based on a null proportion of 1 in 3 (0.33).

Three variables had broad associations (bullying victimization, cannabis use, and lower life satisfaction), being significantly associated with almost every co‐occurring and individual PLE group in all three analyses (the one exception was no significant association between cannabis use and negative symptoms only). For victimization, and life satisfaction, the co‐occurring PLE group had the significantly strongest association in all three analyses, and for cannabis use, the co‐occurring PLE group had the significantly strongest association in two out of the three analyses. Effect sizes were greatest for cannabis use, with ORs of 2.61 to 3.15 for co‐occurring PLE groups.

Family history of schizophrenia had its nominally strongest association with the co‐occurring PLE group in all three analyses. The association was significant for cognitive disorganization plus paranoia or hallucinations, and for negative symptoms plus paranoia or hallucinations. The family history variable was validated by showing significant association with the schizophrenia polygenic risk score (PRS; n=3951, OR=1.73 (95% CI 1.16 to 2.57), p=0.007). We also performed a supplementary analysis of associations between PLE groups and schizophrenia PRS, but there were no statistically significant associations (Tables S4.01–S4.03 in the online supplement). This was probably due to insufficient sample size, as PLEs have shown significant associations in larger analyses that included the current sample (7, 11).

The three variables relating to cognitive function (vocabulary, general cognitive ability, and GCSE score) had their nominally strongest association with an individual PLE (hallucinations, cognitive disorganization, and negative symptoms). Effect sizes were greatest for GCSE score, where the maximal β‐coefficient was 10.45 for negative symptoms occurring in the absence of paranoia or hallucinations.

Obstetric complications had significant associations with PLE groups that included negative symptoms and had its nominally strongest association with negative symptoms co‐occurring with paranoia or hallucinations.

Results for ethnic minority status were inconsistent, being significantly associated with paranoia or hallucinations in the absence of cognitive disorganization, and with negative symptoms in the absence of paranoia or hallucinations.

Paternal age was not significantly associated with any PLE group. As some studies have found the association with schizophrenia to be only with the oldest paternal age‐group (28), we also looked at this variable by 10‐year age bands (Tables S1.05, S2.06, and S3.06 in the online supplement). Each co‐occurring PLE group showed a slight trend towards association with having the oldest fathers (aged 55 years+) but numbers were too small to allow formal analysis.

Patterns of heritability showed variation across the three primary analyses. In the paranoia and hallucinations analysis, only paranoia without hallucinations had significant heritability. In the cognitive disorganization analysis, heritability was nominally highest for cognitive disorganization co‐occurring with paranoia or hallucinations, while cognitive disorganization without paranoia or hallucinations had zero heritability due to unexpectedly higher DZ than MZ concordance. This is more likely to be a chance finding than to be scientifically meaningful because plausible mechanisms for the DZ>MZ pattern are hard to envisage. In the negative symptoms analysis, all three PLE groups had significant heritability, and negative symptoms without paranoia or hallucinations was nominally highest.

### Secondary Analysis

As cognitive disorganization and negative symptoms groups were both associated with all three of the cognitive function variables, we speculated that co‐occurrence of these two PLEs might have stronger associations with cognitive variables than either occurring alone. We therefore conducted a secondary analysis of cognitive disorganization and negative symptoms occurring together or individually.

The summary results are given in Table S5.01 and detailed in Tables S5.02–S5.33 of the online supplement. Co‐occurring cognitive disorganization and negative symptoms had the nominally strongest association with two of the cognitive variables (general cognitive ability, and GCSE score—significantly strongest for GCSE score), while cognitive disorganization without negative symptoms was nominally strongest for vocabulary. Overall, the co‐occurring cognitive disorganization and negative symptoms group had the nominally strongest association with 7 of the 10 variables where at least one PLE group was significantly associated (Table S5.01 in the online supplement: proportion 0.70, p=0.014).

The co‐occurring cognitive disorganization and negative symptoms group had the nominally highest heritability from all of the PLE analyses (0.67 or 67%), although CIs overlapped with the cognitive disorganization only and negative symptoms only groups. The heritability estimate remained similar when based on residualized PLEs after regressing out the effects of sex (0.65, 95%CI 0.23 to 0.77), in male (0.64, 95%CI 0.36 to 0.83) and female (0.67, 95%CI 0.18 to 0.79) twin pairs, and when including opposite‐sex DZ twin pairs (0.65, 95%CI 0.41 to 0.76).

In order to investigate the effects of applying a higher PLE threshold, we conducted the following analyses using a 10% cut‐off. For all PLE combinations, we investigated associations with two prospectively assessed risk factors that showed contrasting patterns of association, general cognitive ability, and victimization. Additionally, for paranoia or hallucinations, and negative symptoms we investigated associations with obstetric complications, and for cognitive disorganization and negative symptoms we investigated twin heritability. The results remained very similar (Tables S6.01–S6.15 in the online supplement). The same associations were statistically significant at 15% and 10% cut‐offs. Among these, the only change in order of nominal effect sizes was that at the 10% cut‐off negative symptoms without paranoia or hallucinations had a marginally stronger association with obstetric complications than negative symptoms plus paranoia or hallucinations (OR 3.04, 95%CI 1.76 to 5.26 vs. 2.87, 95%CI 1.30 to 6.33), reinforcing the previous impression that obstetric complications were most associated with negative symptoms. Also, for most associations at the 10% cut‐off the largest effect size was marginally greater than at the 15% cut‐off.

## Discussion

Three variables—bullying victimization, cannabis use, and lower life satisfaction—had broad associations with most PLE groups and their strongest associations with co‐occurring PLEs, as predicted. These variables might be particularly related to clinical service use, through reduced quality of life or as proximal precipitants of clinical episodes. They may thus contribute to effects seen in adult samples where, relative to individual PLEs, co‐occurring PLEs are associated with increased risk of clinical need (24), schizophrenia (2), and psychotic disorder (21, 22), and other clinically relevant variables, including poorer functioning (20, 25), anxiety symptoms (24) and persistence of PLEs (24). Also, consistent with the current study, co‐occurring PLEs have previously been associated with childhood trauma (24): in that study any lifetime trauma when interviewed at 14–24 years, and in this study with bullying victimization assessed at age 12 years.

Co‐occurring PLEs have previously been associated with more frequent family history of psychosis (23). In the current study, family history of schizophrenia in a first or second degree relative had its nominally strongest association with the co‐occurring PLE group in all three primary comparisons.

The three variables relating to cognitive function had their strongest association with an individual PLE in all three primary analyses. This may have been due to paranoia not being associated with reduced cognitive function (and having a trend in the opposite direction in some analyses) leading to associations for co‐occurring PLE groups that included paranoia being attenuated. To our knowledge, previous studies of co‐occurring PLEs have not investigated cognitive functioning prospectively. However, consistent with our findings, the Avon Longitudinal Study of Parents and Children (ALSPAC) study found only weak evidence for cognitive deficits in adolescence for a psychotic experiences group defined by the presence of hallucination‐ or delusion‐like experiences (36).

Obstetric complications have previously been studied in relation to positive PLEs with mixed results (37, 38), while we found them to be most associated with negative symptoms. Ethnic minority status has been associated with positive PLEs (39). Unfortunately our findings for PLE combinations were inconsistent, and further research is needed in samples that are more ethnically diverse. We found that paternal age was not significantly associated with any PLE group. The ALSPAC study found a non‐significant trend towards an association between positive PLEs at age 12 years and older paternal age (40). As some studies of schizophrenia have found the association only with the oldest fathers (28), and there was a trend consistent with this in the current study, albeit with too small numbers for formal analysis, it is possible that an association with PLEs might be evident in larger samples.

In the secondary analysis of cognitive disorganization and negative symptoms, the group where these two PLEs co‐occurred had the strongest association with schizophrenia‐relevant variables most frequently compared with their individual component PLEs, that is, cognitive disorganization alone and negative symptoms alone. It was also notable that this PLE combination had the highest heritability out of all the PLE groups analyzed (67%). To our knowledge this PLE combination has not been investigated in previous co‐occurrence studies. A negative/disorganized PLE group has been investigated in a past study, but it was predominantly composed of negative symptoms and the component PLEs were not disaggregated (25).

Are there potential implications of these PLE results for clinical diagnoses that are based on the co‐occurrence of symptoms? Where processes underlying PLEs and symptoms are similar, a diagnostic approach might enhance associations with risk factors where most individual symptoms are associated, but could attenuate associations with risk factors where only one type of symptom, for example, negative symptoms, is associated. However, this depends on factors including the relationships between PLEs and symptoms and the processes that underlie the co‐occurrence (22, 23, 24). Further insights may be gained from ongoing research, including longitudinal studies of PLEs and subsequent clinical disorders and their symptom profiles, where relevant risk factors are assessed.

### Limitations

The variables used covered a broader range of prospectively measured relevant variables than previously investigated, but it is possible that the results could have differed if other schizophrenia‐relevant variables were used that were not available in this sample, for example, measures of motor development, other forms of childhood trauma, or other aspects of impairment in life roles. Also results could have differed if alternative questionnaires or interview measures were employed, although the SPEQ is based on well‐established measures that provide reliable and valid PLE subscales.

We defined PLEs in terms of dichotomized scores, that may have reduced power compared to quantitative variables, but gave relative clarity for defining PLE groups and flexibility in allowing both “and” and “or” combinations of PLEs.

Correlations between PLEs and schizophrenia‐relevant variables could be elevated when they had the same rater, and lowered when there were different raters, and the power to detect associations may vary between risk factors because of differences in their prevalence, but the analysis focused on comparing associations for different PLE combinations with each risk factor individually, and then conducting an overall test based on how frequently the co‐occurring PLE group had the strongest association.

We were able to investigate more schizophrenia risk factors than measures of quality of life and functioning. Future research in older participants could extend this to include assessment of, for example, further education, employment, and social relationships.

As part of the development of the PLE measures, twin and singleton comparisons were made and showed no notable differences (3)—twin‐related effects are unlikely but cannot be excluded.

These findings require independent replication, especially as many are reported here for the first time.

## Conclusions

First, we found the patterns of association between PLE combinations and schizophrenia‐relevant variables to vary considerably. Focusing on co‐occurrence enhances some individual PLE associations but obscures others. Second, in terms of which variables are most associated with co‐occurring PLEs, bullying victimization, cannabis use, and lower life satisfaction had the strongest associations with co‐occurring PLEs compared with PLEs in isolation. These variables may be particularly relevant to the association between co‐occurring PLEs and increased risk of clinical disorder and service use. Third, obstetric complications were most associated with negative symptoms, a finding that suggests opportunities for future research into the underlying neurodevelopmental pathways to psychosis vulnerability. Fourth, we found that co‐occurring subjective cognitive disorganization and observed negative symptoms had the at‐least nominally strongest associations across a broad range of schizophrenia‐relevant variables and relatively high heritability. Future research could investigate associations between this PLE combination and other schizophrenia risk factors, and also cumulative exposure to risk factors, and assess how well it predicts onset of schizophrenia.

## Supporting information

Supplementary Material 1

Supplementary Material 2

Supplementary Material 3

Supplementary Material 4

Supplementary Material 5

Supplementary Material 6

Supplementary Material 7
