# Supplementary material for: Psychotic‐Like Experiences in Adolescence Occurring in Combination or Isolation: Associations with Schizophrenia Risk Factors
Source: Psychiatr Res Clin Pract. 2021 Jan 18;3(2):67–75. doi: 10.1176/appi.prcp.20200010 (PMC8609425; doi:10.1176/appi.prcp.20200010)
Supplement: Supplementary file 1 — Supplementary Material 1 [file RCP2-3-67-s006.doc]

Online supplement for Cardno AG et al., Psychotic-like experiences in adolescence occurring in combination or isolation: associations with schizophrenia risk factors

**SUPPLEMENTARY METHODS**

**Psychotic-like experiences measures**

PLEs were assessed using the Specific Psychotic Experiences Questionnaire (SPEQ) at age 16 years (1). The SPEQ assesses specific PLEs as quantitative traits and includes self-report subscales for paranoia (15 items – e.g., ‘Someone has bad intentions towards me’), hallucinations (9 items – e.g., ‘Hear noises or sounds when there is nothing about to explain them’), and cognitive disorganisation (11 items – e.g., ‘Often have difficulties in controlling your thoughts’), and a parent-rated subscale for negative symptoms (10 items - e.g., ‘Seems emotionally “flat”, for example, rarely changes the emotions he/she shows’). SPEQ items were derived from existing scales that were adapted to be suitable for adolescents: the Paranoia Checklist (2); Cardiff Anomalous Perceptions Scale (CAPS) (3); short version of the Oxford-Liverpool Inventory of Feelings and Experiences (O-LIFE) (4); and Scale for the Assessment of Negative Symptoms (SANS) (5). Age appropriateness and validity of items was ensured via obtaining expert clinical opinion and piloting on this age group. The resulting PLE scales were confirmed by principal component analysis and showed good-to-excellent internal consistency (Cronbach’s alpha = 0.77 to 0.93) and test-retest reliability across a nine-month interval (r = 0.65 to 0.74) in this sample (1). The validity of the SPEQ scales is also supported by their associations with family history of schizophrenia (6) and their associations with the schizophrenia polygenic risk score (7, 8).

Spearman correlations between the dichotomised PLEs used in the study ranged from 0.09 (for hallucinations with negative symptoms) to 0.30 (for paranoia with hallucinations).

Overlap between co-occurring PLE groups was as follows: 281 twins scored high on P+H and CD+P/H; 194 twins scored high on P+H and NS+P/H; 230 twins scored high on CD+P/H and NS+P/H; and 91 twins scored high on all three co-occurring PLE groups (note: P = paranoia; H = hallucinations; CD = cognitive disorganisation; P/H = paranoia or hallucinations; NS = negative symptoms).

**Schizophrenia risk factors**

*Family history* (9). Presence/absence of schizophrenia in a first or second degree relative (6). Parents completed a questionnaire when the twins were 16 years old. As this binary variable does not account for the number and age of relatives, we checked that it was associated with the schizophrenia polygenic risk score (PRS) (10). We also performed a supplementary analysis of associations between PLE groups and schizophrenia PRS.

The schizophrenia polygenic risk score (PRS) was calculated by Selzam et al (11), based on the schizophrenia genome-wide association study (GWAS) of Pardiñas et al (12). Following quality control and imputation, genotypic data included 515 100 genotyped or imputed SNPs (info = 1). For more details about genotype processing and quality control, see Selzam et al (13). LDpred (10) was used to calculate the polygenic scores. This is a Bayesian approach, which modifies the summary statistic coefficients based on information on linkage disequilibrium and a prior on the effect size of each SNP. The final PRS is obtained as the sum of the trait-increasing alleles (each variant coded as 0, 1 or 2), weighted by the posterior effect size estimates. LDpred uses levels of assumed fraction of causal markers instead of traditional p-value thresholds. We used an assumed fraction of causal markers of 0.3, which was the optimal level for schizophrenia analyses in the paper that described LDpred (10). Each twin in the analysis was from a separate pair and was of white European ancestry.

We used logistic regression analysis with the PLE group as the dependent variable (e.g. high score on paranoia only vs baseline of high score on neither paranoia nor hallucinations) and PRS as the independent variable, adjusted for the first 10 principal components and genotyping array.

*Older paternal age* (14)*.* Father’s age in years when the twins were born.

*Ethnic minority status* (15). As 93.5% of the sample were of White ethnicity, we combined other ethnic groups to give a binary variable of White/Other ethnicity.

*Obstetric complications* (16). Quantitative score based on the prenatal and neonatal problems scale (PNP), used previously in TEDS (17). This was calculated as the proportion of 17 complications present during pregnancy and the neonatal period: severe stress during pregnancy, amniocentesis, high blood-pressure, diabetes, toxaemia, vaginal bleeding, rubella, slow growth, waters breaking early, labour induced, complications during birth, concerns about twins, twin in special care, returned to hospital as outpatient, returned to hospital overnight, had medical problems, had physical problems.

*Slower developmental milestones* (18). Total vocabulary at age 2 years. Children’s expressive vocabulary was assessed by parent report, using an adaptation of the McArthur‐Bates Communicative Development Index (MCDI) (19, 20). 100 words from the full MCDI were selected and anglicized where necessary (21). Parents were asked to complete a checklist, indicating which words their children could say (disregarding pronunciation errors).

*Lower premorbid IQ* (22, 23). Youth general cognitive ability (g). At age 12 years, the twins participated in web-based testing (24). They underwent two verbal tests, the Wechsler Intelligence Scale for Children (WISC)-III-PI Multiple Choice Information (general knowledge) and Vocabulary Multiple Choice subtests (25), and two non-verbal reasoning tests, the WISC-III-UK Picture Completion (25) and Raven's Standard and Advanced Progressive Matrices (26, 27). A general cognitive ability (g) score with equal weights for the four tests was created by summing their standardized scores (28).

*Bullying victimization* (29). At age 12 years (30), this was assessed by a self-rated questionnaire, the Multidimensional Peer Victimization Scale (31), scored as a total across 16 items encompassing domains of physical abuse, verbal abuse, social manipulation, and property damage. The scale was positively skewed, so a square-root transformation was applied.

*Cannabis use* (32, 33)*.* Participants were asked if they had ever used cannabis at age 16 year assessment (34). This measure was derived from the ALSPAC study.

**Quality of life and functioning variables**

Life satisfaction was measured at age 16 years as the overall mean score on the self-rated Brief Multidimensional Students’ Life Satisfaction Scale (35, 36). The scale was negatively skewed, so a transformation was applied by reversing the scores, taking log_10_, then reversing the scores again.

Functioning was measured at age 16 years using a total point score across all subjects for the UK nationwide examination, the General Certificate of Secondary Education (GCSE), coded from 11 (A*, the highest grade) to 4 (G, the lowest grade) (37).

Spearman correlations between schizophrenia-relevant variables ranged from -0.18 (for bullying victimization aged 12 years with life satisfaction aged 16 years) to 0.46 (for general cognitive ability aged 12 years with GCSE score aged 16 years).

**REFERENCES**

1. Ronald A, Sieradzka D, Cardno AG, et al: Characterization of psychotic experiences in adolescence using the specific psychotic experiences questionnaire: findings from a study of 5000 16-year-old twins. Schizophr Bull 2014; 40:868-877

2. Freeman D, Garety PA, Bebbington PE, et al: Psychological investigation of the structure of paranoia in a non-clinical population. Br J Psychiatry 2005; 186:427-435

3. Bell V, Halligan PW, Ellis HD: The Cardiff Anomalous Perceptions Scale (CAPS): a new validated measure of anomalous perceptual experience. Schizophr Bull 2006; 32:366-377

4. Mason O, Linney Y, Claridge G: Short scales for measuring schizotypy. Schizophr Res 2005; 78:293-296

5. Andreasen NC: The Scale for the Assessment of Negative symptoms (SANS). Iowa City, IA, University of Iowa, 1984

6. Zavos HM, Freeman D, Haworth CM, et al: Consistent etiology of severe, frequent psychotic experiences and milder, less frequent manifestations: a twin study of specific psychotic experiences in adolescence. JAMA Psychiatry 2014; 71:1049- 1057

7. Pain O, Dudbridge F, Cardno AG, et al: Genome-wide analysis of adolescent psychotic-like experiences shows genetic overlap with psychiatric disorders. Am J Med Genet B Neuropsychiatr Genet 2018; 177:416-425

8. Ronald A, Pain O: A systematic review of genome-wide research on psychotic experiences and negative symptom traits: new revelations and implications for psychiatry. Hum Mol Genet 2018; 27:R136-R152

9. Lichtenstein P, Yip BH, Bjork C, et al: Common genetic determinants of schizophrenia and bipolar disorder in Swedish families: a population-based study. Lancet 2009; 373:234-239

10. Vilhjalmsson BJ, Yang J, Finucane HK, et al: Modeling linkage disequilibrium increases accuracy of polygenic risk scores. Am J Hum Genet 2015; 97:576-592

11. Selzam S, Ritchie SJ, Pingault JB, et al: Comparing within- and between-family polygenic score prediction. Am J Hum Genet 2019; 105:351-363

12. Pardinas AF, Holmans P, Pocklington AJ, et al: Common schizophrenia alleles are enriched in mutation-intolerant genes and in regions under strong background selection. Nat Genet 2018; 50:381-389

13. Selzam S, McAdams TA, Coleman JRI, et al: Evidence for gene-environment correlation in child feeding: Links between common genetic variation for BMI in children and parental feeding practices. PLoS Genet 2018; 14:e1007757

14. de Kluiver H, Buizer-Voskamp JE, Dolan CV, et al: Paternal age and psychiatric disorders: A review. Am J Med Genet B Neuropsychiatr Genet 2017; 174:202-213

15. Kirkbride JB, Hameed Y, Ioannidis K, et al: Ethnic minority status, age-at-immigration and psychosis risk in rural environments: evidence from the SEPEA study. Schizophr Bull 2017; 43:1251-1261

16. Cannon M, Jones PB, Murray RM: Obstetric complications and schizophrenia: historical and meta-analytic review. Am J Psychiatry 2002; 159:1080-1092

17. Ronald A, Happe F, Dworzynski K, et al: Exploring the relation between prenatal and neonatal complications and later autistic-like features in a representative community sample of twins. Child Dev 2010; 81:166-182

18. Jones P, Rodgers B, Murray R, et al: Child development risk factors for adult schizophrenia in the British 1946 birth cohort. Lancet 1994; 344:1398-1402

19. Fenson L, Dale PS, Reznick JS, et al: Variability in early communicative development. Monogr Soc Res Child Dev 1994; 59:1-173; discussion 174-185

20. Fenson L, Pethick S, Renda C, et al: Short-form versions of the MacArthur communicative development inventories. Appl Psycholinguist 2000; 21:95-116

21. Hayiou-Thomas ME, Dale PS, Plomin R: The etiology of variation in language skills changes with development: a longitudinal twin study of language from 2 to 12 years. Dev Sci 2012; 15:233-249

22. Woodberry KA, Giuliano AJ, Seidman LJ: Premorbid IQ in schizophrenia: a meta- analytic review. Am J Psychiatry 2008; 165:579-587

23. Zammit S, Allebeck P, David AS, et al: A longitudinal study of premorbid IQ Score and risk of developing schizophrenia, bipolar disorder, severe depression, and other nonaffective psychoses. Arch Gen Psychiatry 2004; 61:354-360

24. Haworth CM, Harlaar N, Kovas Y, et al: Internet cognitive testing of large samples needed in genetic research. Twin Res Hum Genet 2007; 10:554-563

25. Wechsler D: Wechsler Intelligence Scale for Children—Third Edition UK (WISC- IIIUK) Manual. London, The Psychological Corporation, 1992

26. Raven JC, Court JH, Raven J: Manual for Raven's Progressive Matrices and Vocabulary Scales. Oxford, Oxford University Press, 1996

27. Raven JC, Court JH, Raven J: Manual for Raven's Progressive Matrices. London, HK Lewis, 1998

28. Haworth CM, Wright MJ, Luciano M, et al: The heritability of general cognitive ability increases linearly from childhood to young adulthood. Mol Psychiatry 2010; 15:1112- 1120

29. Varese F, Smeets F, Drukker M, et al: Childhood adversities increase the risk of psychosis: a meta-analysis of patient-control, prospective- and cross-sectional cohort studies. Schizophr Bull 2012; 38:661-671

30. Shakoor S, McGuire P, Cardno AG, et al: A shared genetic propensity underlies experiences of bullying victimization in late childhood and self-rated paranoid thinking in adolescence. Schizophr Bull 2015; 41:754-763

31. Mynard H, Joseph S: Development of the multidimensional peer‐victimization scale. Aggr Behav 2000; 26:169-178

32. Marconi A, Di Forti M, Lewis CM, et al: Meta-analysis of the association between the level of cannabis use and risk of psychosis. Schizophr Bull 2016; 42:1262-1269

33. Moore TH, Zammit S, Lingford-Hughes A, et al: Cannabis use and risk of psychotic or affective mental health outcomes: a systematic review. Lancet 2007; 370:319-328

34. Shakoor S, Zavos HM, McGuire P, et al: Psychotic experiences are linked to cannabis use in adolescents in the community because of common underlying environmental risk factors. Psychiatry Res 2015; 227:144-151

35. Hosang GM, Cardno AG, Freeman D, et al: Characterization and structure of hypomania in a British nonclinical adolescent sample. J Affect Disord 2017; 207:228- 235

36. Seligson JL, Huebner ES, Valois RF: Preliminary validation of the Brief Multidimensional Students’ Life Satisfaction Scale (BMSLSS). Soc Indic Res 2003; 61:121–145

37. Shakeshaft NG, Trzaskowski M, McMillan A, et al: Strong genetic influence on a UK nationwide test of educational achievement at the end of compulsory education at age 16. PloS One 2013; 8:e80341
