# Supplementary material for: Psychotic‐Like Experiences in Adolescence Occurring in Combination or Isolation: Associations with Schizophrenia Risk Factors
Source: Psychiatr Res Clin Pract. 2021 Jan 18;3(2):67–75. doi: 10.1176/appi.prcp.20200010 (PMC8609425; doi:10.1176/appi.prcp.20200010)
Supplement: Supplementary file 2 — Supplementary Material 2 [file RCP2-3-67-s005.doc]

Online supplement for Cardno AG et al., Psychotic-like experiences in adolescence occurring in combination or isolation: associations with schizophrenia risk factors

**SUPPLEMENTARY RESULTS (1): Analysis of Paranoia and Hallucinations**

| **CONTENTS** | **Page** |
| --- | --- |
| Family history of schizophrenia | 2 |
| Paternal age | 3 |
| Ethnicity | 4 |
| Obstetric complications | 5 |
| Vocabulary | 6 |
| General cognitive ability | 7 |
| Bullying victimization | 8 |
| Cannabis use | 9 |
| Life satisfaction | 10 |
| GCSE score | 11 |
| Twin heritability | 12 |

**Family history of schizophrenia in a 1^st^ or 2^nd^ degree relative**

| **Table S1.01. Cross tabulation of paranoia and hallucinations by family history of schizophrenia** | | | | | |
| --- | --- | --- | --- | --- | --- |
|  | | | Family history of schizophrenia | | Total |
|  |  |  | No | Yes |  |
| Paranoia and hallucinations top 15% | High on neither P nor H | Count | 6950 | 190 | 7140 |
|  |  | % | 97.3% | 2.7% | 100.0% |
|  | high on P but not H | Count | 772 | 29 | 801 |
|  |  | % | 96.4% | 3.6% | 100.0% |
|  | high on H but not P | Count | 954 | 22 | 976 |
|  |  | % | 97.7% | 2.3% | 100.0% |
|  | high on both P and H | Count | 600 | 24 | 624 |
|  |  | % | 96.2% | 3.8% | 100.0% |
| Total | | Count | 9276 | 265 | 9541 |
|  |  | % | 97.2% | 2.8% | 100.0% |

Note: P, paranoia; H, hallucinations.

**Table S1.02. Logistic regression analysis of paranoia and hallucinations with family history of schizophrenia^a^**

| Comparison | n | OR (95% CI) | P-value |
| --- | --- | --- | --- |
| P only vs neither | 7941 | 1.36 (0.85 to 2.19) | 0.20 |
| H only vs neither | 8116 | 0.84 (0.53 to 1.33) | 0.46 |
| P+H vs neither | 7764 | 1.46 (0.88 to 2.41) | 0.14 |

Note: ^a^Generalized estimating equations (GEE) approach, adjusted for birth order, sex, and age ~16 years when returned psychotic-like experiences questionnaires. OR, odds ratio; P, paranoia; H, hallucinations.

**Paternal age**

| **Table S1.03. Descriptive statistics of paranoia and hallucinations with paternal age** | | | | | | |
| --- | --- | --- | --- | --- | --- | --- |
| Age in years of natural father at time of birth of twins | | | | | | |
| Paranoia and hallucinations top 15% | N | Mean | Std. Deviation | Median | Minimum | Maximum |
| High on neither P nor H | 6651 | 33.7210 | 5.55120 | 33.1691 | 17.94 | 61.23 |
| high on P but not H | 753 | 33.7981 | 5.64917 | 33.2539 | 18.86 | 56.61 |
| high on H but not P | 905 | 33.5943 | 5.92426 | 33.0815 | 16.94 | 61.23 |
| high on both P and H | 563 | 33.7057 | 5.74189 | 33.0075 | 18.95 | 59.67 |
| Total | 8872 | 33.7136 | 5.61010 | 33.1691 | 16.94 | 61.23 |

Note: P, paranoia; H, hallucinations.

**Table S1.04. Logistic regression analysis of paranoia and hallucinations with paternal age^a^**

| Comparison | n | OR (95% CI) | P-value |
| --- | --- | --- | --- |
| P only vs neither | 7404 | 1.003 (0.989 to 1.018) | 0.66 |
| H only vs neither | 7556 | 0.996 (0.982 to 1.011) | 0.63 |
| P+H vs neither | 7214 | 1.000 (0.983 to 1.017) | 0.96 |

Note: ^a^Generalized estimating equations (GEE) approach, adjusted for birth order, sex, and age ~16 years when returned psychotic-like experiences questionnaires. OR, odds ratio; P, paranoia; H, hallucinations.

| **Table S1.05. Cross tabulation of paranoia and hallucinations by paternal age in 10 year bands** | | | | | | | | |
| --- | --- | --- | --- | --- | --- | --- | --- | --- |
|  | | | Age of father in 10 year bands | | | | | Total |
|  |  |  | <25y | 25-34y | 35-44y | 45-54y | 55y+ |  |
|  | High on neither P nor H | Count | 277 | 3870 | 2280 | 212 | 12 | 6651 |
|  |  | % | 4.2% | 58.2% | 34.3% | 3.2% | 0.2% | 100.0% |
|  | high on P but not H | Count | 27 | 436 | 258 | 31 | 1 | 753 |
|  |  | % | 3.6% | 57.9% | 34.3% | 4.1% | 0.1% | 100.0% |
|  | high on H but not P | Count | 49 | 512 | 307 | 35 | 2 | 905 |
|  |  | % | 5.4% | 56.6% | 33.9% | 3.9% | 0.2% | 100.0% |
|  | high on both P and H | Count | 24 | 329 | 188 | 18 | 4 | 563 |
|  |  | % | 4.3% | 58.4% | 33.4% | 3.2% | 0.7% | 100.0% |
| Total | | Count | 377 | 5147 | 3033 | 296 | 19 | 8872 |
|  |  | % | 4.2% | 58.0% | 34.2% | 3.3% | 0.2% | 100.0% |

Note: y, years; P, paranoia; H, hallucinations.

**Ethnic minority status**

| **Table S1.06. Cross tabulation of paranoia and hallucinations by ethnicity** | | | | | |
| --- | --- | --- | --- | --- | --- |
|  | | | Ethnicity of twins (1=White, 0=Other) | | Total |
|  |  |  | 0 | 1 |  |
| Paranoia and hallucinations top 15% | High on neither P nor H | Count | 450 | 6753 | 7203 |
|  |  | % | 6.2% | 93.8% | 100.0% |
|  | high on P but not H | Count | 49 | 762 | 811 |
|  |  | % | 6.0% | 94.0% | 100.0% |
|  | high on H but not P | Count | 78 | 908 | 986 |
|  |  | % | 7.9% | 92.1% | 100.0% |
|  | high on both P and H | Count | 48 | 580 | 628 |
|  |  | % | 7.6% | 92.4% | 100.0% |
| Total | | Count | 625 | 9003 | 9628 |
|  |  | % | 6.5% | 93.5% | 100.0% |

Note: P, paranoia; H, hallucinations.

**Table S1.07. Logistic regression analysis of paranoia and hallucinations with ethnicity^a^**

| Comparison | n | OR (95% CI) | P-value |
| --- | --- | --- | --- |
| P only vs neither | 8014 | 0.95 (0.68 to 1.32) | 0.74 |
| H only vs neither | 8189 | 1.27 (0.95 to 1.70) | 0.10 |
| P+H vs neither | 7831 | 1.23 (0.87 to 1.73) | 0.24 |

Note: ^a^Generalized estimating equations (GEE) approach, adjusted for birth order, sex, and age ~16 years when returned psychotic-like experiences questionnaires. OR, odds ratio; P, paranoia; H, hallucinations.

**Obstetric complications**

| **Table S1.08. Descriptive statistics of paranoia and hallucinations with obstetric complications** | | | | | | |
| --- | --- | --- | --- | --- | --- | --- |
| Obstetric complications score | | | | | | |
| Paranoia and hallucinations top 15% | N | Mean | Std. Deviation | Median | Minimum | Maximum |
| High on neither P nor H | 7150 | .2086 | .14105 | .1765 | .00 | .92 |
| high on P but not H | 810 | .2111 | .14148 | .1765 | .00 | .89 |
| high on H but not P | 976 | .2042 | .13067 | .1765 | .00 | .82 |
| high on both P and H | 622 | .2196 | .14744 | .1875 | .00 | .90 |
| Total | 9558 | .2091 | .14051 | .1765 | .00 | .92 |

Note: P, paranoia; H, hallucinations.

**Table S1.09. Logistic regression analysis of paranoia and hallucinations with obstetric complications^a^ GEE (adjusted for birth order, sex, age ~16 years when returned PLE questionnaires)**

| Comparison | n | OR (95% CI) | P-value |
| --- | --- | --- | --- |
| P only vs neither | 7960 | 1.15 (0.68 to 1.97) | 0.61 |
| H only vs neither | 8126 | 0.83 (0.51 to 1.35) | 0.45 |
| P+H vs neither | 7772 | 1.79 (0.98 to 3.28) | 0.06 |

Note: ^a^Generalized estimating equations (GEE) approach, adjusted for birth order, sex, and age ~16 years when returned psychotic-like experiences questionnaires. OR, odds ratio; P, paranoia; H, hallucinations.

**Vocabulary age 2 years**

| **Table S1.10. Paranoia and hallucinations with vocabulary age 2 years descriptive statistics** | | | | | | |
| --- | --- | --- | --- | --- | --- | --- |
| Vocabulary total score | | | | | | |
| Paranoia and hallucinations top 15% | N | Mean | Std. Deviation | Median | Minimum | Maximum |
| High on neither P nor H | 3763 | 48.79 | 24.512 | 47.00 | 0 | 100 |
| high on P but not H | 455 | 50.17 | 25.282 | 49.00 | 1 | 100 |
| high on H but not P | 549 | 46.66 | 25.228 | 45.00 | 2 | 100 |
| high on both P and H | 310 | 49.40 | 25.030 | 46.50 | 2 | 100 |
| Total | 5077 | 48.72 | 24.699 | 47.00 | 0 | 100 |

Note: P, paranoia; H, hallucinations.

**Table S1.11. Logistic regression analysis of paranoia and hallucinations with vocabulary age 2 years^a^**

| Comparison | n | OR (95% CI) | P-value |
| --- | --- | --- | --- |
| P only vs neither | 4038 | 1.003 (0.998 to 1.007) | 0.21 |
| H only vs neither | 4114 | 0.996 (0.991 to 1.000) | 0.047 |
| P+H vs neither | 3900 | 1.000 (0.995 to 1.005) | 1.00 |

Note: ^a^Generalized estimating equations (GEE) approach, adjusted for birth order, sex, age ~16 years when returned psychotic-like experiences questionnaires, age ~2 years when vocabulary assessed, and socioeconomic status at 1st contact. OR, odds ratio; P, paranoia; H, hallucinations.

**General cognition age 12 years**

| **Table S1.12. Descriptive statistics of paranoia and hallucinations with general cognition age 12 years** | | | | | | |
| --- | --- | --- | --- | --- | --- | --- |
| General cognition standardised score | | | | | | |
| Paranoia and hallucinations top 15% | N | Mean | Std. Deviation | Median | Minimum | Maximum |
| High on neither P nor H | 4632 | .061021 | .9946610 | .119245 | -3.6803 | 2.8111 |
| high on P but not H | 525 | .088564 | 1.0135399 | .119725 | -2.6455 | 2.5497 |
| high on H but not P | 604 | -.077316 | .9747186 | -.031964 | -3.4714 | 2.1627 |
| high on both P and H | 408 | .012920 | .9412353 | -.004388 | -2.9085 | 2.2000 |
| Total | 6169 | .046639 | .9916202 | .094054 | -3.6803 | 2.8111 |

Note: P, paranoia; H, hallucinations.

**Table S1.13. Logistic regression analysis of paranoia and hallucinations with general cognition age 12 years^a^**

| Comparison | n | OR (95% CI) | P-value |
| --- | --- | --- | --- |
| P only vs neither | 4948 | 1.053 (0.945 to 1.174) | 0.351 |
| H only vs neither | 5019 | 0.919 (0.835 to 1.013) | 0.089 |
| P+H vs neither | 4833 | 1.021 (0.909 to 1.147) | 0.723 |

Note: ^a^Generalized estimating equations (GEE) approach, adjusted for birth order, sex, age ~16 years when returned psychotic-like experiences questionnaires, and socioeconomic status at 1st contact. OR, odds ratio; P, paranoia; H, hallucinations.

**Bullying victimization age 12 years**

| **Table S1.14. Descriptive statistics of paranoia and hallucinations with bullying victimization age 12 years** | | | | | | |
| --- | --- | --- | --- | --- | --- | --- |
| Victimization total score (square root transformation) | | | | | | |
| Paranoia and hallucinations top 15% | N | Mean | Std. Deviation | Median | Minimum | Maximum |
| High on neither P nor H | 5771 | 2.1659 | 1.43008 | 2.2361 | .00 | 5.66 |
| high on P but not H | 674 | 2.8082 | 1.42569 | 2.8284 | .00 | 5.66 |
| high on H but not P | 759 | 2.5552 | 1.39036 | 2.4495 | .00 | 5.66 |
| high on both P and H | 502 | 3.0358 | 1.41333 | 3.1623 | .00 | 5.66 |
| Total | 7706 | 2.3171 | 1.45141 | 2.2361 | .00 | 5.66 |

Note: P, paranoia; H, hallucinations.

**Table S1.15. Logistic regression analysis of paranoia and hallucinations with bullying victimization age 12 years^a^**

| Comparison | n | OR (95% CI) | P-value |
| --- | --- | --- | --- |
| P only vs neither | 6146 | 1.418 (1.330 to 1.511) | <0.001 |
| H only vs neither | 6225 | 1.236 (1.165 to 1.310) | <0.001 |
| P+H vs neither | 5988 | 1.591 (1.472 to 1.719) | <0.001 |

Note: ^a^Generalized estimating equations (GEE) approach, adjusted for birth order, sex, age ~16 years when returned psychotic-like experiences questionnaires, and socioeconomic status at 1st contact. OR, odds ratio; P, paranoia; H, hallucinations.

**Table S1.16. Post hoc analysis - logistic regression analysis of paranoia and hallucinations with bullying victimization age 12 years^a^**

| Comparison | n | OR (95% CI) | P-value |
| --- | --- | --- | --- |
| P+H vs P only | 1122 | 1.124 (1.029 to 1.228) | 0.010 |

Note: ^a^Generalized estimating equations (GEE) approach, adjusted for birth order, sex, age ~16 years when returned psychotic-like experiences questionnaires, and socioeconomic status at 1st contact. OR, odds ratio; P, paranoia; H, hallucinations.

**Cannabis use by age 16 years**

| **Table S1.17. Cross tabulation of paranoia and hallucinations by cannabis use** | | | | | |
| --- | --- | --- | --- | --- | --- |
|  | | | Ever tried cannabis by age 16 years (0=no, 1=yes) | | Total |
|  |  |  | 0 | 1 |  |
| Paranoia and hallucinations top 15% | High on neither P nor H | Count | 5073 | 457 | 5530 |
|  |  | % | 91.7% | 8.3% | 100.0% |
|  | high on P but not H | Count | 468 | 81 | 549 |
|  |  | % | 85.2% | 14.8% | 100.0% |
|  | high on H but not P | Count | 623 | 106 | 729 |
|  |  | % | 85.5% | 14.5% | 100.0% |
|  | high on both P and H | Count | 386 | 82 | 468 |
|  |  | % | 82.5% | **17.5%** | 100.0% |
| Total | | Count | 6550 | 726 | 7276 |
|  |  | % | 90.0% | 10.0% | 100.0% |

Note: P, paranoia; H, hallucinations.

**Table S1.18. Logistic regression analysis of paranoia and hallucinations with cannabis use by age 16 years^a^**

| Comparison | n | OR (95% CI) | P-value |
| --- | --- | --- | --- |
| P only vs neither | 5756 | 1.888 (1.426 to 2.499) | <0.001 |
| H only vs neither | 5922 | 2.044 (1.591 to 2.625) | <0.001 |
| P+H vs neither | 5682 | 2.612 (1.960 to 3.481) | <0.001 |

Note: ^a^Generalized estimating equations (GEE) approach, adjusted for birth order, sex, age ~16 years when returned psychotic-like experiences questionnaires, and socioeconomic status at 1st contact. OR, odds ratio; P, paranoia; H, hallucinations.

.

**Table S1.19. Post hoc analysis - logistic regression analysis of paranoia and hallucinations with cannabis use by age 16 years^a^**

| Comparison | n | OR (95% CI) | P-value |
| --- | --- | --- | --- |
| P+H vs H only | 1122 | 1.299 (0.923 to 1.829) | 0.134 |

Note: ^a^Generalized estimating equations (GEE) approach, adjusted for birth order, sex, age ~16 years when returned psychotic-like experiences questionnaires, and socioeconomic status at 1st contact. OR, odds ratio; P, paranoia; H, hallucinations.

**Life satisfaction age 16 years**

| **Table S1.20. Descriptive statistics of paranoia and hallucinations with life satisfaction age 16 years** | | | | | | |
| --- | --- | --- | --- | --- | --- | --- |
| Life satisfaction score (transformed: reverse score then log_10_ then reverse again) | | | | | | |
| Paranoia and hallucinations top 15% | N | Mean | Std. Deviation | Median | Minimum | Maximum |
| High on neither P nor H | 7203 | 1.5713 | .17587 | 1.5868 | 1.00 | 1.85 |
| high on P but not H | 812 | 1.3825 | .16829 | 1.3729 | 1.03 | 1.85 |
| high on H but not P | 984 | 1.4723 | .17403 | 1.4820 | 1.02 | 1.85 |
| high on both P and H | 626 | 1.3563 | .17370 | 1.3494 | 1.00 | 1.85 |
| Total | 9625 | 1.5313 | .18973 | 1.5490 | 1.00 | 1.85 |

Note: P, paranoia; H, hallucinations.

**Table S1.21. Linear regression analysis of paranoia and hallucinations with life satisfaction age 16 years^a^**

| Comparison | β (95% CI) | P-value |
| --- | --- | --- |
| P only vs neither | -0.186 (-0.199 to -0.173) | <0.001 |
| H only vs neither | -0.097 (-0.110 to -0.085) | <0.001 |
| P+H vs neither | -0.210 (-0.225 to -0.195) | <0.001 |

Note: ^a^Generalized estimating equations (GEE) approach, adjusted for birth order, sex, age ~16 years when returned psychotic-like experiences questionnaires, and socioeconomic status at 1st contact (n=9119). P, paranoia; H, hallucinations.

**Table S1.22. Post hoc analysis - linear regression analysis of paranoia and hallucinations with life satisfaction age 16 years^a^**

| Comparison | n | β (95% CI) | P-value |
| --- | --- | --- | --- |
| P+H vs P only | 1359 | -0.022 (-0.040 to -0.003) | 0.023 |

Note: ^a^Generalized estimating equations (GEE) approach, adjusted for birth order, sex, age ~16 years when returned psychotic-like experiences questionnaires, and socioeconomic status at 1st contact. P, paranoia; H, hallucinations.

**GCSE exams total point score age 16 years**

| **Table S1.23. Descriptive statistics of paranoia and hallucinations with GCSE exams total point score age 16 years** | | | | | | |
| --- | --- | --- | --- | --- | --- | --- |
| GCSE exams total point score | | | | | | |
| Paranoia and hallucinations top 15% | N | Mean | Std. Deviation | Median | Minimum | Maximum |
| High on neither P nor H | 6340 | 86.6958 | 24.98048 | 90.0000 | .00 | 180.50 |
| high on P but not H | 708 | 86.1900 | 25.71926 | 89.0000 | .00 | 148.00 |
| high on H but not P | 860 | 82.5041 | 24.54537 | 85.0000 | .00 | 149.00 |
| high on both P and H | 552 | 82.6141 | 24.84981 | 85.0000 | .00 | 146.00 |
| Total | 8460 | 85.9611 | 25.03350 | 89.0000 | .00 | 180.50 |

Note: GCSE, General Certificate of Secondary Education; P, paranoia; H, hallucinations.

**Table S1.24. Linear regression analysis of paranoia and hallucinations with GCSE exams total point score age 16 years^a^**

| PLE group | β (95% CI) | P-value |
| --- | --- | --- |
| P only | -0.061 (-1.933 to 1.812) | 0.949 |
| H only | -2.881 (-4.559 to -1.203) | 0.001 |
| P+H | -2.858 (-4.902 to -0.814) | 0.006 |

Note: ^a^Generalized estimating equations (GEE) approach, adjusted for birth order, sex, age ~16 years when returned psychotic-like experiences questionnaires, and socioeconomic status at 1st contact (n=8056). P, paranoia; H, hallucinations.

**Twin modelling**

**Table S1.25. Probandwise concordances**

| PLE group | MZ concordance (%) | SS DZ concordance (%) |
| --- | --- | --- |
| P only | 80/289 (27.7%) | 38/251 (15.1%) |
| H only | 94/353 (26.6%) | 60/297 (20.2%) |
| P+H | 50/207 (24.2%) | 38/191 (19.9%) |

Note: PLE, psychotic-like experiences; MZ, monozygotic; SS DZ, same-sex dizygotic; P, paranoia; H, hallucinations.

**Table S1.26. Tetrachoric correlations^a^**

| PLE group | MZ (95% CI) | SS DZ (95% CI) |
| --- | --- | --- |
| P only | 0.46 (0.34 to 0.56) | 0.21 (0.06 to 0.35) |
| H only | 0.39 (0.28 to 0.49) | 0.28 (0.15 to 0.41) |
| P+H | 0.47 (0.33 to 0.59) | 0.38 (0.23 to 0.52) |

Note: ^a^1724 MZ pairs, 1547 SS DZ pairs. Calculated with the same threshold for both twins and both zygosities as this was best-fitting. PLE, psychotic-like experiences; MZ, monozygotic; SS DZ, same-sex dizygotic; P, paranoia; H, hallucinations.

**Table S1.27. Parameter estimates for the ACE model^a^**

| PLE group | a^2^ (95% CI) | c^2^ (95% CI) | e^2^ (95% CI) |
| --- | --- | --- | --- |
| P only | 0.45 (0.13 to 0.55) | 0.00 (0.00 to 0.27) | 0.55 (0.45 to 0.66) |
| H only | 0.21 (0.00 to 0.49) | 0.18 (0.00 to 0.41) | 0.61 (0.51 to 0.72) |
| P+H | 0.17 (0.00 to 0.56) | 0.29 (0.00 to 0.51) | 0.53 (0.41 to 0.66) |

Note: ^a^1724 MZ pairs, 1547 SS DZ pairs. ACE model, twin analysis model including additive genetic, common environmental, and individual-specific environmental effects; PLE, psychotic-like experiences; a^2^, c^2^, e^2^, variance in liability due to additive genetic effects (heritability – also symbolised by h^2^), common environmental effects and individual-specific environmental effects; P, paranoia; H, hallucinations; MZ, monozygotic; SS DZ, same-sex dizygotic.
