# Supplementary material for: Psychotic‐Like Experiences in Adolescence Occurring in Combination or Isolation: Associations with Schizophrenia Risk Factors
Source: Psychiatr Res Clin Pract. 2021 Jan 18;3(2):67–75. doi: 10.1176/appi.prcp.20200010 (PMC8609425; doi:10.1176/appi.prcp.20200010)
Supplement: Supplementary file 3 — Supplementary Material 3 [file RCP2-3-67-s004.doc]

Online supplement for Cardno AG et al., Psychotic-like experiences in adolescence occurring in combination or isolation: associations with schizophrenia risk factors

**SUPPLEMENTARY RESULTS (2): Analysis of Cognitive Disorganisation and (Paranoia or Hallucinations)**

| **CONTENTS** | **Page** |
| --- | --- |
| Family history of schizophrenia | 2 |
| Paternal age | 3 |
| Ethnicity | 4 |
| Obstetric complications | 5 |
| Vocabulary | 6 |
| General cognitive ability | 7 |
| Bullying victimization | 8 |
| Cannabis use | 9 |
| Life satisfaction | 10 |
| GCSE score | 11 |
| Twin heritability | 12 |

**Family history of schizophrenia in a 1^st^ or 2^nd^** degree relative

| **Table S2.01. Cross tabulation of cognitive disorganisation and (paranoia or hallucinations) by family history of schizophrenia** | | | | | |
| --- | --- | --- | --- | --- | --- |
|  | | | Family history of schizophrenia | | Total |
|  |  |  | No | Yes |  |
| Cognitive disorganisation and (paranoia or hallucinations) top 15% | None of CD, P or H | Count | 6391 | 169 | 6560 |
|  |  | % | 97.4% | 2.6% | 100.0% |
|  | CD & (not P nor H) | Count | 548 | 20 | 568 |
|  |  | % | 96.5% | 3.5% | 100.0% |
|  | (P or H) & not CD | Count | 1610 | 45 | 1655 |
|  |  | % | 97.3% | 2.7% | 100.0% |
|  | CD & (P or H) | Count | 711 | 30 | 741 |
|  |  | % | 96.0% | 4.0% | 100.0% |
| Total | | Count | 9260 | 264 | 9524 |
|  |  | % | 97.2% | 2.8% | 100.0% |

Note: Sz, schizophrenia; CD, cognitive disorganisation; P, paranoia; H, hallucinations.

**Table S2.02. Logistic regression analysis of cognitive disorganisation and (paranoia or hallucinations) with family history of schizophrenia^a^**

| Comparison | n | OR (95% CI) | P-value |
| --- | --- | --- | --- |
| CD only vs neither | 6768 | 1.488 (0.887 to 2.496) | 0.132 |
| (P or H) only vs neither | 7800 | 1.000 (0.675 to 1.481) | 1.000 |
| CD+(P or H) vs neither | 6930 | 1.717 (1.074 to 2.746) | 0.024 |

Note: ^a^Generalized estimating equations (GEE) approach, adjusted for birth order, sex, age ~16 years when returned psychotic-like experience questionnaires, and socioeconomic status at 1st contact. OR, odds ratio; CD, cognitive disorganisation; P, paranoia; H, hallucinations.

**Table S2.03. Post hoc analysis - logistic regression analysis of cognitive disorganisation and (paranoia or hallucinations) with family history of schizophrenia^a^**

| Comparison | n | OR (95% CI) | P-value |
| --- | --- | --- | --- |
| CD+(P or H) vs CD only | 1228 | 1.143 (0.610 to 2.138) | 0.677 |

Note: ^a^Generalized estimating equations (GEE) approach, adjusted for birth order, sex, age ~16 years when returned psychotic-like experience questionnaires, and socioeconomic status at 1st contact. OR, odds ratio; CD, cognitive disorganisation; P, paranoia; H, hallucinations.

**Paternal age**

| **Table S2.04. Descriptive statistics of cognitive disorganisation and (paranoia or hallucinations) with paternal age** | | | | | | |
| --- | --- | --- | --- | --- | --- | --- |
| Age in years of natural father at time of birth of twins | | | | | | |
| CD and (P or H)  top 15% | N | Mean | Std. Deviation | Median | Minimum | Maximum |
| None of CD, P or H | 6116 | 33.7269 | 5.56265 | 33.1513 | 17.94 | 59.67 |
| CD & (not P nor H) | 524 | 33.6755 | 5.45960 | 33.4264 | 18.45 | 61.23 |
| (P or H) & not CD | 1544 | 33.7407 | 5.68445 | 33.1869 | 16.94 | 59.67 |
| CD & (P or H) | 673 | 33.5887 | 6.01122 | 33.0212 | 16.94 | 61.23 |
| Total | 8857 | 33.7158 | 5.61246 | 33.1691 | 16.94 | 61.23 |

Note: CD, cognitive disorganisation; P, paranoia; H, hallucinations.

**Table S2.05. Logistic regression analysis of cognitive disorganisation and (paranoia or hallucinations) with paternal age^a^**

| Comparison | n | OR (95% CI) | P-value |
| --- | --- | --- | --- |
| CD only vs neither | 6640 | 0.999 (0.982 to 1.016) | 0.92 |
| (P or H) only vs neither | 7660 | 1.001 (0.990 to 1.012) | 0.88 |
| CD+(P or H) vs neither | 6789 | 0.996 (0.980 to 1.013) | 0.66 |

Note: ^a^Generalized estimating equations (GEE) approach, adjusted for birth order, sex, and age ~16 years when returned psychotic-like experience questionnaires. OR, odds ratio; CD, cognitive disorganisation; P, paranoia; H, hallucinations.

| **Table S2.06. Cross tabulation of cognitive disorganisation and (paranoia or hallucinations) by paternal age in 10 year bands** | | | | | | | | |
| --- | --- | --- | --- | --- | --- | --- | --- | --- |
|  | | | Age of father in 10 year bands | | | | | Total |
|  |  |  | <25y | 25-34y | 35-44y | 45-54y | 55y+ |  |
|  | None of CD, P or H | Count | 255 | 3555 | 2094 | 201 | 11 | 6116 |
|  |  | % | 4.2% | 58.1% | 34.2% | 3.3% | 0.2% | 100.0% |
|  | CD & (not P nor H) | Count | 22 | 307 | 183 | 11 | 1 | 524 |
|  |  | % | 4.2% | 58.6% | 34.9% | 2.1% | 0.2% | 100.0% |
|  | (P or H) & not CD | Count | 63 | 886 | 533 | 59 | 3 | 1544 |
|  |  | % | 4.1% | 57.4% | 34.5% | 3.8% | 0.2% | 100.0% |
|  | CD & (P or H) | Count | 37 | 389 | 218 | 25 | 4 | 673 |
|  |  | % | 5.5% | 57.8% | 32.4% | 3.7% | 0.6% | 100.0% |
| Total | | Count | 377 | 5137 | 3028 | 296 | 19 | 8857 |
|  |  | % | 4.3% | 58.0% | 34.2% | 3.3% | 0.2% | 100.0% |

Note: y, years; CD, cognitive disorganisation; P, paranoia; H, hallucinations.

**Ethnic minority status**

| **Table S2.07. Cross tabulation of cognitive disorganisation and (paranoia or hallucinations) by ethnicity** | | | | | |
| --- | --- | --- | --- | --- | --- |
|  | | | Ethnicity of twins (1=White, 0=Other) | | Total |
|  |  |  | 0 | 1 |  |
| CD and (P or H) top 15% | None of CD, P or H | Count | 407 | 6214 | 6621 |
|  |  | % | 6.1% | 93.9% | 100.0% |
|  | CD & (not P nor H) | Count | 43 | 527 | 570 |
|  |  | % | 7.5% | 92.5% | 100.0% |
|  | (P or H) & not CD | Count | 133 | 1539 | 1672 |
|  |  | % | 8.0% | 92.0% | 100.0% |
|  | CD & (P or H) | Count | 41 | 707 | 748 |
|  |  | % | 5.5% | 94.5% | 100.0% |
| Total | | Count | 624 | 8987 | 9611 |
|  |  | % | 6.5% | 93.5% | 100.0% |

Note: CD, cognitive disorganisation; P, paranoia; H, hallucinations.

**Table S2.08. Logistic regression analysis of cognitive disorganisation and (paranoia or hallucinations) with ethnicity^a^**

| Comparison | n | OR (95% CI) | P-value |
| --- | --- | --- | --- |
| CD only vs neither | 6828 | 1.218 (0.845 to 1.755) | 0.291 |
| (P or H) only vs neither | 7871 | 1.293 (1.007 to 1.661) | 0.044 |
| CD+(P or H) vs neither | 6995 | 0.751 (0.495 to 1.139) | 0.178 |

Note: ^a^Generalized estimating equations (GEE) approach, adjusted for birth order, sex, age ~16 years when returned psychotic-like experience questionnaires, and socioeconomic status at 1st contact. OR, odds ratio; CD, cognitive disorganisation; P, paranoia; H, hallucinations.

**Obstetric complications**

| **Table S2.09. Descriptive statistics of cognitive disorganisation and (paranoia or hallucinations) with obstetric complications** | | | | | | |
| --- | --- | --- | --- | --- | --- | --- |
| Obstetric complications score | | | | | | |
| CD and (P or H)  top 15% | N | Mean | Std. Deviation | Median | Minimum | Maximum |
| None of Cog Dis, P or H | 6571 | .2096 | .14181 | .1765 | .00 | .92 |
| Cog Dis & (not P nor H) | 567 | .1961 | .13063 | .1765 | .00 | .64 |
| (P or H) & not Cog Dis | 1657 | .2077 | .13871 | .1765 | .00 | .90 |
| Cog Dis & (P or H) | 746 | .2171 | .13912 | .1875 | .00 | .82 |
| Total | 9541 | .2091 | .14046 | .1765 | .00 | .92 |

Note: CD, cognitive disorganisation; P, paranoia; H, hallucinations.

**Table S2.10. Logistic regression analysis of cognitive disorganisation and (paranoia or hallucinations) with obstetric complications^a^**

| Comparison | n | OR (95% CI) | P-value |
| --- | --- | --- | --- |
| CD only vs neither | 7138 | 0.53 (0.28 to 1.00) | 0.050 |
| (P or H) only vs neither | 8228 | 0.92 (0.61 to 1.37) | 0.67 |
| CD+(P or H) vs neither | 7317 | 1.57 (0.91 to 2.73) | 0.11 |

Note: ^a^Generalized estimating equations (GEE) approach, adjusted for birth order, sex, and age ~16 years when returned psychotic-like experience questionnaires. OR, odds ratio; CD, cognitive disorganisation; P, paranoia; H, hallucinations.

**Vocabulary age 2 years**

| **Table S2.11. Descriptive statistics of cognitive disorganisation and (paranoia or hallucinations) with vocabulary age 2 years** | | | | | | |
| --- | --- | --- | --- | --- | --- | --- |
| Vocabulary total score | | | | | | |
| CD and (P or H)  top 15% | N | Mean | Std. Deviation | Median | Minimum | Maximum |
| None of CD, P or H | 3451 | 49.13 | 24.557 | 48.00 | 0 | 100 |
| CD & (not P nor H) | 305 | 45.11 | 23.896 | 44.00 | 0 | 100 |
| (P or H) & not CD | 905 | 48.86 | 25.499 | 47.00 | 1 | 100 |
| CD & (P or H) | 408 | 47.81 | 24.669 | 46.50 | 2 | 100 |
| Total | 5069 | 48.73 | 24.710 | 47.00 | 0 | 100 |

Note: CD, cognitive disorganisation; P, paranoia; H, hallucinations.

**Table S2.12. Logistic regression analysis of cognitive disorganisation and (paranoia or hallucinations) with vocabulary age 2 years^a^**

| Comparison | n | OR (95% CI) | P-value |
| --- | --- | --- | --- |
| CD only vs neither | 3594 | 0.992 (0.987 to 0.997) | 0.002 |
| (P or H) only vs neither | 4170 | 0.999 (0.996 to 1.003) | 0.76 |
| CD+(P or H) vs neither | 3694 | 0.996 (0.992 to 1.001) | 0.14 |

Note: ^a^Generalized estimating equations (GEE) approach, adjusted for birth order, sex, age ~16 years when returned psychotic-like experience questionnaires, age ~2 years when vocabulary assessed, and socioeconomic status at 1st contact. OR, odds ratio; CD, cognitive disorganisation; P, paranoia; H, hallucinations.

**General cognition age 12 years**

| **Table S2.13. Descriptive statistics of cognitive disorganisation and (paranoia or hallucinations) with general cognition age 12 years** | | | | | | |
| --- | --- | --- | --- | --- | --- | --- |
| General cognition standardised score | | | | | | |
| CD and (P or H)  top 15% | N | Mean | Std. Deviation | Median | Minimum | Maximum |
| None of CD, P or H | 4282 | .079527 | .9894712 | .134908 | -3.5933 | 2.8111 |
| CD & (not P nor H) | 343 | -.175745 | 1.0321138 | -.141405 | -3.6803 | 2.2359 |
| (P or H) & not CD | 1074 | .066969 | .9789474 | .099717 | -3.4714 | 2.5497 |
| CD & (P or H) | 459 | -.144774 | .9734205 | -.130093 | -3.0308 | 2.0566 |
| Total | 6158 | .046399 | .9918274 | .093941 | -3.6803 | 2.8111 |

Note: CD, cognitive disorganisation; P, paranoia; H, hallucinations.

**Table S2.14. Logistic regression analysis of cognitive disorganisation and (paranoia or hallucinations) with general cognition age 12 years^a^**

| Comparison | n | OR (95% CI) | P-value |
| --- | --- | --- | --- |
| CD only vs neither | 4437 | 0.749 (0.657 to 0.854) | <0.001 |
| (P or H) only vs neither | 5141 | 1.022 (0.944 to 1.107) | 0.591 |
| CD+(P or H) vs neither | 4546 | 0.851 (0.762 to 0.949) | 0.004 |

Note: : ^a^Generalized estimating equations (GEE) approach, adjusted for birth order, sex, age ~16 years when returned psychotic-like experience questionnaires, and socioeconomic status at 1st contact. OR, odds ratio; CD, cognitive disorganisation; P, paranoia; H, hallucinations.

**Bullying victimization age 12 years**

| **Table S2.15. Descriptive statistics of cognitive disorganisation and (paranoia or hallucinations) with bullying victimization age 12 years** | | | | | | |
| --- | --- | --- | --- | --- | --- | --- |
| Victimization total score (square root transformation) | | | | | | |
| CD and (P or H)  top 15% | N | Mean | Std. Deviation | Median | Minimum | Maximum |
| None of CD, P or H | 5310 | 2.1327 | 1.42516 | 2.0000 | .00 | 5.66 |
| CD & (not P nor H) | 450 | 2.5503 | 1.42865 | 2.6458 | .00 | 5.66 |
| (P or H) & not CD | 1338 | 2.6949 | 1.43621 | 2.8284 | .00 | 5.66 |
| CD & (P or H) | 592 | 2.9422 | 1.36715 | 3.0000 | .00 | 5.66 |
| Total | 7690 | 2.3173 | 1.45103 | 2.2361 | .00 | 5.66 |

Note: CD, cognitive disorganisation; P, paranoia; H, hallucinations.

**Table S2.16. Logistic regression analysis of cognitive disorganisation and (paranoia or hallucinations) with bullying victimization age 12 years^a^**

| Comparison | n | OR (95% CI) | P-value |
| --- | --- | --- | --- |
| CD only vs neither | 5495 | 1.259 (1.167 to 1.359) | <0.001 |
| (P or H) only vs neither | 6354 | 1.347 (1.283 to 1.415) | <0.001 |
| CD+(P or H) vs neither | 5635 | 1.579 (1.473 to 1.693) | <0.001 |

Note: ^a^Generalized estimating equations (GEE) approach, adjusted for birth order, sex, age ~16 years when returned psychotic-like experience questionnaires, and socioeconomic status at 1st contact. OR, odds ratio; CD, cognitive disorganisation; P, paranoia; H, hallucinations.

**Table S2.17. Post hoc analysis - logistic regression analysis of cognitive disorganisation and (paranoia or hallucinations) with bullying victimization age 12 years^a^**

| Comparison | n | OR (95% CI) | P-value |
| --- | --- | --- | --- |
| CD+(P or H) vs (P or H) only | 1837 | 1.162 (1.078 to 1.252) | <0.001 |

Note: ^a^Generalized estimating equations (GEE) approach, adjusted for birth order, sex, age ~16 years when returned psychotic-like experience questionnaires, and socioeconomic status at 1st contact. OR, odds ratio; CD, cognitive disorganisation; P, paranoia; H, hallucinations.

**Cannabis use** by age 16 years

| **Table S2.18. Cross tabulation of cognitive disorganisation and (paranoia or hallucinations) by cannabis use** | | | | | |
| --- | --- | --- | --- | --- | --- |
|  | | | Ever tried cannabis by age 16 years (0=no, 1=yes) | | Total |
|  |  |  | 0 | 1 |  |
| CD and (P or H) top 15% | None of CD, P or H | Count | 4692 | 404 | 5096 |
|  |  | % | 92.1% | 7.9% | 100.0% |
|  | CD & (not P nor H) | Count | 373 | 52 | 425 |
|  |  | % | 87.8% | 12.2% | 100.0% |
|  | (P or H) & not CD | Count | 1033 | 167 | 1200 |
|  |  | % | 86.1% | 13.9% | 100.0% |
|  | CD & (P or H) | Count | 441 | 102 | 543 |
|  |  | % | 81.2% | 18.8% | 100.0% |
| Total | | Count | 6539 | 725 | 7264 |
|  |  | % | 90.0% | 10.0% | 100.0% |

Note: CD, cognitive disorganisation; P, paranoia; H, hallucinations.

**Table S2.19. Logistic regression analysis of cognitive disorganisation and (paranoia or hallucinations) with cannabis use by age 16 years^a^**

| Comparison | n | OR (95% CI) | P-value |
| --- | --- | --- | --- |
| CD only vs neither | 5232 | 1.895 (1.352 to 2.655) | <0.001 |
| (P or H) only vs neither | 5956 | 1.947 (1.575 to 2.408) | <0.001 |
| CD+(P or H) vs neither | 5342 | 3.149 (2.406 to 4.123) | <0.001 |

Note: ^a^Generalized estimating equations (GEE) approach, adjusted for birth order, sex, age ~16 years when returned psychotic-like experience questionnaires, and socioeconomic status at 1st contact. OR, odds ratio; CD, cognitive disorganisation; P, paranoia; H, hallucinations.

**Table S2.20. Post hoc analysis - logistic regression analysis of cognitive disorganisation and (paranoia or hallucinations) with cannabis use by age 16 years^a^**

| Comparison | n | OR (95% CI) | P-value |
| --- | --- | --- | --- |
| CD+(P or H) vs (P or H) only | 1634 | 1.561 (1.161 to 2.099) | 0.003 |

Note: ^a^Generalized estimating equations (GEE) approach, adjusted for birth order, sex, age ~16 years when returned psychotic-like experience questionnaires, and socioeconomic status at 1st contact. OR, odds ratio; CD, cognitive disorganisation; P, paranoia; H, hallucinations.

**Life satisfaction age 16 years**

| **Table S2.21. Descriptive statistics of cognitive disorganisation and (paranoia or hallucinations) with life satisfaction age 16 years** | | | | | | |
| --- | --- | --- | --- | --- | --- | --- |
| Life satisfaction score (transformed: reverse score then log_10_ then reverse again) | | | | | | |
| CD and (P or H)  top 15% | N | Mean | Std. Deviation | Median | Minimum | Maximum |
| None of CD, P or H | 6625 | 1.5830 | .17068 | 1.5868 | 1.04 | 1.85 |
| CD & (not P nor H) | 572 | 1.4349 | .17753 | 1.4380 | 1.00 | 1.85 |
| (P or H) & not CD | 1669 | 1.4465 | .17584 | 1.4521 | 1.00 | 1.85 |
| CD & (P or H) | 752 | 1.3359 | .16274 | 1.3059 | 1.02 | 1.85 |
| Total | 9618 | 1.5312 | .18972 | 1.5490 | 1.00 | 1.85 |

Note: CD, cognitive disorganisation; P, paranoia; H, hallucinations.

**Table S2.22. Linear regression analysis of cognitive disorganisation and (paranoia or hallucinations) with life satisfaction age 16 years^a^**

| Comparison | β (95% CI) | P-value |
| --- | --- | --- |
| CD only vs neither | -0.145 (-0.161 to -0.129) | <0.001 |
| (P or H) only vs neither | -0.134 (-0.144 to -0.124) | <0.001 |
| CD+(P or H) vs neither | -0.246 (-0.259 to -0.233) | <0.001 |

Note: ^a^Generalized estimating equations (GEE) approach, adjusted for birth order, sex, age ~16 years when returned psychotic-like experience questionnaires, and socioeconomic status at 1st contact (n=9112). CD, cognitive disorganisation; P, paranoia; H, hallucinations.

**Table S2.23. Post hoc analysis - linear regression analysis of cognitive disorganisation and (paranoia or hallucinations) with life satisfaction age 16 years^a^**

| Comparison | n | β (95% CI) | P-value |
| --- | --- | --- | --- |
| Cog Dis+(P or H) vs Cog Dis only | 1242 | -0.101 (-0.120 to -0.082) | <0.001 |

Note: ^a^Generalized estimating equations (GEE) approach, adjusted for birth order, sex, age ~16 years when returned psychotic-like experience questionnaires, and socioeconomic status at 1st contact. CD, cognitive disorganisation; P, paranoia; H, hallucinations.

**GCSE exams total point score age 16 years**

| **Table S2.24. Descriptive statistics of cognitive disorganisation and (paranoia or hallucinations) with GCSE exams total point score age 16 years** | | | | | | |
| --- | --- | --- | --- | --- | --- | --- |
| GCSE exams total point score | | | | | | |
| CD and (P or H)  top 15% | N | Mean | Std. Deviation | Median | Minimum | Maximum |
| None of CD, P or H | 5847 | 87.4406 | 24.68496 | 90.5000 | .00 | 180.50 |
| CD & (not P nor H) | 485 | 78.0072 | 26.67681 | 82.5000 | 5.00 | 137.00 |
| (P or H) & not CD | 1451 | 86.2688 | 24.57789 | 89.0000 | .00 | 149.00 |
| CD & (P or H) | 665 | 78.3368 | 25.32115 | 81.0000 | .00 | 144.00 |
| Total | 8448 | 85.9811 | 25.02654 | 89.0000 | .00 | 180.50 |

Note: GCSE, General Certificate of Secondary Education; CD, cognitive disorganisation; P, paranoia; H, hallucinations.

**Table S2.25. Linear regression analysis of cognitive disorganisation and (paranoia or hallucinations) with GCSE exams total point score age 16 years^a^**

| Comparison | β (95% CI) | P-value |
| --- | --- | --- |
| Cog Dis vs neither | -8.609 (-10.876 to -6.343) | <0.001 |
| (P or H) vs neither | -0.495 (-1.825 to 0.836) | 0.466 |
| Cog Dis+(P or H) vs neither | -7.334 (-9.323 to -5.345) | <0.001 |

Note: ^a^Generalized estimating equations (GEE) approach, adjusted for birth order, sex, age ~16 years when returned psychotic-like experience questionnaires, and socioeconomic status at 1st contact (n=8045). CD, cognitive disorganisation; P, paranoia; H, hallucinations.

**Twin modelling**

**Table S2.26. Probandwise concordances**

| PLE group | MZ concordance (%) | SS DZ concordance (%) |
| --- | --- | --- |
| CD only | 22/187 (11.8%) | 42/212 (19.8%) |
| (P or H) only | 200/579 (34.5%) | 142/508 (28.0%) |
| CD+(P or H) | 84/266 (31.6%) | 38/226 (16.8%) |

Note: PLE, psychotic-like experiences; MZ, monozygotic; SS DZ, same-sex dizygotic; CD, cognitive disorganisation; P, paranoia; H, hallucinations.

**Table S2.27. Tetrachoric correlations^a^**

| PLE group | MZ (95% CI) | SS DZ (95% CI) |
| --- | --- | --- |
| CD only | 0.23 (0.05 to 0.40) | 0.35 (0.20 to 0.48) |
| (P or H) only | 0.39 (0.30 to 0.48) | 0.27 (0.17 to 0.37) |
| CD+(P or H) | 0.54 (0.43 to 0.63) | 0.28 (0.12 to 0.42) |

Note: ^a^1719 MZ pairs, 1539 SS DZ pairs. Calculated with the same threshold for both twins and both zygosities as this was best-fitting. PLE, psychotic-like experiences; MZ, monozygotic; SS DZ, same-sex dizygotic; CD, cognitive disorganisation; P, paranoia; H, hallucinations.

**Table S2.28. Parameter estimates for the ACE model^a^**

| PLE group | a^2^ (95% CI) | c^2^ (95% CI) | e^2^ (95% CI) |
| --- | --- | --- | --- |
| CD only | 0.00 (0.00 to 0.00) | 0.30 (0.09 to 0.40) | 0.70 (0.58 to 0.81) |
| (P or H) only | 0.24 (0.00 to 0.47) | 0.15 (0.00 to 0.37) | 0.61 (0.52 to 0.70) |
| Cog Dis+(P or H) | 0.52 (0.16 to 0.63) | 0.02 (0.00 to 0.32) | 0.46 (0.37 to 0.57) |

Note: ^a^1719 MZ pairs, 1539 SS DZ pairs. ACE model, twin analysis model including additive genetic, common environmental, and individual-specific environmental effects; PLE, psychotic-like experiences; a^2^, c^2^, e^2^, variance in liability due to additive genetic effects (heritability – also symbolised by h^2^), common environmental effects and individual-specific environmental effects; CD, cognitive disorganisation; P, paranoia; H, hallucinations; MZ, monozygotic; SS DZ, same-sex dizygotic.
