# Supplementary material for: Psychotic‐Like Experiences in Adolescence Occurring in Combination or Isolation: Associations with Schizophrenia Risk Factors
Source: Psychiatr Res Clin Pract. 2021 Jan 18;3(2):67–75. doi: 10.1176/appi.prcp.20200010 (PMC8609425; doi:10.1176/appi.prcp.20200010)
Supplement: Supplementary file 4 — Supplementary Material 4 [file RCP2-3-67-s007.doc]

Online supplement for Cardno AG et al., Psychotic-like experiences in adolescence occurring in combination or isolation: associations with schizophrenia risk factors

**SUPPLEMENTARY RESULTS (3): Analysis of Negative Symptoms and (Paranoia or Hallucinations)**

| **CONTENTS** | **Page** |
| --- | --- |
| Family history of schizophrenia | 2 |
| Paternal age | 3 |
| Ethnicity | 4 |
| Obstetric complications | 5 |
| Vocabulary | 6 |
| General cognitive ability | 7 |
| Bullying victimization | 8 |
| Cannabis use | 9 |
| Life satisfaction | 10 |
| GCSE score | 11 |
| Twin heritability | 12 |

**Family history of schizophrenia in a 1^st^ or 2^nd^ degree relative**

| **Table S3.01. Cross tabulation of negative symptoms and (paranoia or hallucinations) by family history of schizophrenia** | | | | | |
| --- | --- | --- | --- | --- | --- |
|  | | | Family history of schizophrenia | | Total |
|  |  |  | No | Yes |  |
| NS and (P or H)  top 15% | None of NS, P or H | Count | 5973 | 152 | 6125 |
|  |  | % | 97.5% | 2.5% | 100.0% |
|  | NS & (not P nor H) | Count | 970 | 38 | 1008 |
|  |  | % | 96.2% | 3.8% | 100.0% |
|  | (P or H) & not NS | Count | 1785 | 51 | 1836 |
|  |  | % | 97.2% | 2.8% | 100.0% |
|  | NS & (P or H) | Count | 539 | 24 | 563 |
|  |  | % | 95.7% | 4.3% | 100.0% |
| Total | | Count | 9267 | 265 | 9532 |
|  |  | % | 97.2% | 2.8% | 100.0% |

Note: Sz, schizophrenia; NS, negative symptoms; P, paranoia; H, hallucinations.

**Table S3.02. Logistic regression analysis of negative symptoms and (paranoia or hallucinations) with family history of schizophrenia^a^**

| Comparison | n | OR (95% CI) | P-value |
| --- | --- | --- | --- |
| NS only vs neither | 6772 | 1.546 (0.970 to 2.464) | 0.067 |
| (P or H) only vs neither | 7594 | 1.095 (0.739 to 1.621) | 0.651 |
| NS+(P or H) vs neither | 6362 | 1.777 (1.002 to 3.152) | 0.049 |

Note: ^a^Generalized estimating equations (GEE) approach, adjusted for birth order, sex, age ~16 years when returned psychotic-like experience questionnaires, and socioeconomic status at 1st contact. OR, odds ratio; NS, negative symptoms; P, paranoia; H, hallucinations.

**Table S3.03. Post hoc analysis – logistic regression analysis of negative symptoms and (paranoia or hallucinations) with family history of schizophrenia^a^**

| Comparison | n | OR (95% CI) | P-value |
| --- | --- | --- | --- |
| NS+(P or H) vs NS only | 1440 | 1.081 (0.576 to 2.026) | 0.809 |

Note: ^a^Generalized estimating equations (GEE) approach, adjusted for birth order, sex, age ~16 years when returned psychotic-like experience questionnaires, and socioeconomic status at 1st contact. OR, odds ratio; NS, negative symptoms; P, paranoia; H, hallucinations.

**Paternal age**

| **Table S3.04. Descriptive statistics of negative symptoms and (paranoia or hallucinations) with paternal age** | | | | | | |
| --- | --- | --- | --- | --- | --- | --- |
| Age in years of natural father at time of birth of twins | | | | | | |
| NS and (P or H)  top 15% | N | Mean | Std. Deviation | Median | Minimum | Maximum |
| None of NS, P or H | 5702 | 33.7555 | 5.51498 | 33.1718 | 18.45 | 61.23 |
| NS & (not P nor H) | 903 | 33.5339 | 5.82256 | 33.1280 | 17.94 | 57.95 |
| (P or H) & not NS | 1714 | 33.7391 | 5.66777 | 33.2772 | 16.94 | 59.67 |
| NS & (P or H) | 494 | 33.4487 | 6.02565 | 32.6982 | 18.95 | 61.23 |
| Total | 8813 | 33.7124 | 5.60642 | 33.1691 | 16.94 | 61.23 |

Note: NS, negative symptoms; P, paranoia; H, hallucinations.

**Table S3.05. Logistic regression analysis of negative symptoms and (paranoia or hallucinations) with paternal age^a^**

| Comparison | n | OR (95% CI) | P-value |
| --- | --- | --- | --- |
| NS only vs neither | 6605 | 0.992 (0.976 to 1.008) | 0.310 |
| (P or H) only vs neither | 7416 | 1.000 (0.989 to 1.011) | 0.989 |
| NS+(P or H) vs neither | 6196 | 0.990 (0.969 to 1.012) | 0.362 |

Note: ^a^Generalized estimating equations (GEE) approach, adjusted for birth order, sex, and age ~16 years when returned psychotic-like experience questionnaires. OR, odds ratio; NS, negative symptoms; P, paranoia; H, hallucinations.

| **Table S3.06. Cross tabulation of negative symptoms and (paranoia or hallucinations) by paternal age in 10 year bands** | | | | | | | | |
| --- | --- | --- | --- | --- | --- | --- | --- | --- |
|  | | | Age of father in 10 year bands | | | | | Total |
|  |  |  | <25y | 25-34y | 35-44y | 45-54y | 55y+ |  |
|  | None of NS, P or H | Count | 228 | 3325 | 1955 | 184 | 10 | 5702 |
|  |  | % | 4.0% | 58.3% | 34.3% | 3.2% | 0.2% | 100.0% |
|  | NS & (not P nor H) | Count | 48 | 515 | 310 | 28 | 2 | 903 |
|  |  | % | 5.3% | 57.0% | 34.3% | 3.1% | 0.2% | 100.0% |
|  | (P or H) & not NS | Count | 75 | 982 | 590 | 65 | 2 | 1714 |
|  |  | % | 4.4% | 57.3% | 34.4% | 3.8% | 0.1% | 100.0% |
|  | NS & (P or H) | Count | 24 | 288 | 160 | 19 | 3 | 494 |
|  |  | % | 4.9% | 58.3% | 32.4% | 3.8% | 0.6% | 100.0% |
| Total | | Count | 375 | 5110 | 3015 | 296 | 17 | 8813 |
|  |  | % | 4.3% | 58.0% | 34.2% | 3.4% | 0.2% | 100.0% |

Note: y, years; NS, negative symptoms; P, paranoia; H, hallucinations.

**Ethnic minority status**

Note: NS, negative symptoms; P, paranoia; H, hallucinations.

| **Table S3.07. Cross tabulation of negative symptoms and (paranoia or hallucinations) by ethnicity** | | | | | |
| --- | --- | --- | --- | --- | --- |
|  | | | Ethnicity of twins (1=White, 0=Other) | | Total |
|  |  |  | 0 | 1 |  |
| NS and (P or H)  top 15% | None of NS, P or H | Count | 357 | 5778 | 6135 |
|  |  | % | 5.8% | 94.2% | 100.0% |
|  | NS & (not P nor H) | Count | 91 | 925 | 1016 |
|  |  | % | 9.0% | 91.0% | 100.0% |
|  | (P or H) & not NS | Count | 129 | 1713 | 1842 |
|  |  | % | 7.0% | 93.0% | 100.0% |
|  | NS & (P or H) | Count | 43 | 525 | 568 |
|  |  | % | 7.6% | 92.4% | 100.0% |
| Total | | Count | 620 | 8941 | 9561 |
|  |  | % | 6.5% | 93.5% | 100.0% |

**Table S3.08. Logistic regression analysis of negative symptoms and (paranoia or hallucinations) with ethnicity^a^**

| Comparison | n | OR (95%CI) | P-value |
| --- | --- | --- | --- |
| NS only vs neither | 6790 | 1.660 (1.219 to 2.260) | 0.001 |
| (P or H) only vs neither | 7612 | 1.213 (0.938 to 1.568) | 0.141 |
| NS+(P or H) vs neither | 6377 | 1.128 (0.735 to 1.731) | 0.582 |

Note: ^a^Generalized estimating equations (GEE) approach, adjusted for birth order, sex, age ~16 years when returned psychotic-like experience questionnaires, and socioeconomic status at 1st contact. OR, odds ratio; NS, negative symptoms; P, paranoia; H, hallucinations.

**Obstetric complications**

| **Table S3.09. Descriptive statistics of negative symptoms and (paranoia or hallucinations) with obstetric complications** | | | | | | |
| --- | --- | --- | --- | --- | --- | --- |
| Obstetric complications score | | | | | | |
| NS and (P or H)  top 15% | N | Mean | Std. Deviation | Median | Minimum | Maximum |
| None of NS, P or H | 6097 | .2069 | .14067 | .1765 | .00 | .92 |
| NS & (not P nor H) | 1003 | .2195 | .14306 | .1875 | .00 | .83 |
| (P or H) & not NS | 1829 | .2070 | .13962 | .1765 | .00 | .90 |
| NS & (P or H) | 564 | .2222 | .13701 | .2000 | .00 | .82 |
| Total | 9493 | .2092 | .14058 | .1765 | .00 | .92 |

Note: NS, negative symptoms; P, paranoia; H, hallucinations.

**Table S3.10. Logistic regression analysis of negative symptoms and (paranoia or hallucinations) with obstetric complications^a^**

| Comparison | n | OR (95% CI) | P-value |
| --- | --- | --- | --- |
| NS only vs neither | 6747 | 2.053 (1.198 to 3.519) | 0.009 |
| (P or H) only vs neither | 7571 | 1.085 (0.717 to 1.642) | 0.700 |
| NS+(P or H) vs neither | 6341 | 2.378 (1.234 to 4.584) | 0.010 |

Note: ^a^Generalized estimating equations (GEE) approach, adjusted for birth order, sex, age ~16 years when returned psychotic-like experience questionnaires, and socioeconomic status at 1st contact. OR, odds ratio; NS, negative symptoms; P, paranoia; H, hallucinations.

**Table S3.11. Post hoc analysis - logistic regression analysis of negative symptoms and (paranoia or hallucinations) with obstetric complications^a^**

| Comparison | n | OR (95% CI) | P-value |
| --- | --- | --- | --- |
| NS+(P or H) vs NS only | 1432 | 1.166 (0.510 to 2.665) | 0.715 |

Note: ^a^Generalized estimating equations (GEE) approach, adjusted for birth order, sex, age ~16 years when returned psychotic-like experience questionnaires, and socioeconomic status at 1st contact. OR, odds ratio; NS, negative symptoms; P, paranoia; H, hallucinations.

.

**Vocabulary age 2 years**

| **Table S3.12. Descriptive statistics of negative symptoms and (paranoia or hallucinations) with vocabulary age 2 years** | | | | | | |
| --- | --- | --- | --- | --- | --- | --- |
| Vocabulary total score | | | | | | |
| NS and (P or H)  top 15% | N | Mean | Std. Deviation | Median | Minimum | Maximum |
| None of NS, P or H | 3219 | 49.35 | 24.451 | 48.00 | 1 | 100 |
| NS & (not P nor H) | 511 | 45.29 | 24.701 | 44.00 | 0 | 100 |
| (P or H) & not NS | 1017 | 48.91 | 25.097 | 48.00 | 1 | 100 |
| NS & (P or H) | 289 | 47.39 | 25.659 | 46.00 | 2 | 100 |
| Total | 5036 | 48.73 | 24.703 | 47.00 | 0 | 100 |

Note: NS, negative symptoms; P, paranoia; H, hallucinations.

**Table S3.13. Logistic regression analysis of negative symptoms and (paranoia or hallucinations) with vocabulary age 2 years^a^**

| Comparison | n | OR (95% CI) | P-value |
| --- | --- | --- | --- |
| NS only vs neither | 3568 | 0.995 (0.990 to 0.9997) | 0.037 |
| (P or H) only vs neither | 4066 | 0.999 (0.995 to 1.002) | 0.391 |
| NS+(P or H) vs neither | 3359 | 0.998 (0.992 to 1.004) | 0.540 |

Note: ^a^Generalized estimating equations (GEE) approach, adjusted for birth order, sex, age ~16 years when returned psychotic-like experience questionnaires, age ~2 years when vocabulary assessed, and socioeconomic status at 1st contact. OR, odds ratio; NS, negative symptoms; P, paranoia; H, hallucinations.

**General cognition age 12 years**

| **Table S3.14. Descriptive statistics of negative symptoms and (paranoia or hallucinations) with general cognition age 12 years** | | | | | | |
| --- | --- | --- | --- | --- | --- | --- |
| General cognition standardised score | | | | | | |
| NS and (P or H)  top 15% | N | Mean | Std. Deviation | Median | Minimum | Maximum |
| None of NS, P or H | 4021 | .105822 | .9762599 | .160360 | -3.5134 | 2.8111 |
| NS & (not P nor H) | 590 | -.250967 | 1.0673656 | -.179800 | -3.6803 | 2.3563 |
| (P or H) & not NS | 1175 | .069521 | .9639422 | .094450 | -3.4714 | 2.5497 |
| NS & (P or H) | 353 | -.207216 | 1.0009565 | -.164157 | -3.0308 | 2.0997 |
| Total | 6139 | .046584 | .9916288 | .093206 | -3.6803 | 2.8111 |

Note: NS, negative symptoms; P, paranoia; H, hallucinations.

**Table S3.15. Logistic regression analysis of negative symptoms and (paranoia or hallucinations) with general cognition age 12 years^a^**

| Comparison | n | OR (95% CI) | P-value |
| --- | --- | --- | --- |
| NS only vs neither | 4423 | 0.711 (0.639 to 0.791) | <0.001 |
| (P or H) only vs neither | 5002 | 0.988 (0.913 to 1.068) | 0.761 |
| NS+(P or H) vs neither | 4196 | 0.816 (0.713 to 0.934) | 0.003 |

Note: ^a^Generalized estimating equations (GEE) approach, adjusted for birth order, sex, age ~16 years when returned psychotic-like experience questionnaires, and socioeconomic status at 1st contact. OR, odds ratio; NS, negative symptoms; P, paranoia; H, hallucinations.

**Bullying victimization age 12 years**

| **Table S3.16. Descriptive statistics of negative symptoms and (paranoia or hallucinations) with bullying victimization age 12 years** | | | | | | |
| --- | --- | --- | --- | --- | --- | --- |
| Victimization total score (square root transformation) | | | | | | |
| NS and (P or H)  top 15% | N | Mean | Std. Deviation | Median | Minimum | Maximum |
| None of NS, P or H | 4962 | 2.1247 | 1.42530 | 2.0000 | .00 | 5.66 |
| NS & (not P nor H) | 779 | 2.4248 | 1.43435 | 2.4495 | .00 | 5.66 |
| (P or H) & not NS | 1483 | 2.7060 | 1.42564 | 2.8284 | .00 | 5.66 |
| NS & (P or H) | 442 | 2.9673 | 1.39316 | 3.0000 | .00 | 5.66 |
| Total | 7666 | 2.3163 | 1.45157 | 2.2361 | .00 | 5.66 |

Note: NS, negative symptoms; P, paranoia; H, hallucinations.

**Table S3.17. Logistic regression analysis of negative symptoms and (paranoia or hallucinations) with bullying victimization age 12 years^a^**

| Comparison | n | OR (95% CI) | P-value |
| --- | --- | --- | --- |
| NS only vs neither | 5477 | 1.112 (1.046 to 1.183) | 0.001 |
| (P or H) only vs neither | 6183 | 1.369 (1.305 to 1.436) | <0.001 |
| NS+(P or H) vs neither | 5163 | 1.510 (1.394 to 1.636) | <0.001 |

Note: ^a^Generalized estimating equations (GEE) approach, adjusted for birth order, sex, age ~16 years when returned psychotic-like experience questionnaires, and socioeconomic status at 1st contact. OR, odds ratio; NS, negative symptoms; P, paranoia; H, hallucinations.

**Table S3.18. Post hoc analysis - logistic regression analysis of negative symptoms and (paranoia or hallucinations) with bullying victimization age 12 years^a^**

| Comparison | n | OR (95% CI) | P-value |
| --- | --- | --- | --- |
| NS+(P or H) vs (P or H) only | 1832 | 1.116 (1.026 to 1.214) | 0.011 |

Note: ^a^Generalized estimating equations (GEE) approach, adjusted for birth order, sex, age ~16 years when returned psychotic-like experience questionnaires, and socioeconomic status at 1st contact. OR, odds ratio; NS, negative symptoms; P, paranoia; H, hallucinations.

**Cannabis use by age 16 years**

| **Table S3.19. Cross tabulation of negative symptoms and (paranoia or hallucinations) by cannabis use** | | | | | |
| --- | --- | --- | --- | --- | --- |
|  | | | Ever tried cannabis by age 16 years (0=no, 1=yes) | | Total |
|  |  |  | 0 | 1 |  |
| NS and (P or H)  top 15% | None of NS, P or H | Count | 4332 | 384 | 4716 |
|  |  | % | 91.9% | 8.1% | 100.0% |
|  | NS & (not P nor H) | Count | 707 | 69 | 776 |
|  |  | % | 91.1% | 8.9% | 100.0% |
|  | (P or H) & not NS | Count | 1125 | 183 | 1308 |
|  |  | % | 86.0% | 14.0% | 100.0% |
|  | NS & (P or H) | Count | 342 | 84 | 426 |
|  |  | % | 80.3% | 19.7% | 100.0% |
| Total | | Count | 6506 | 720 | 7226 |
|  |  | % | 90.0% | 10.0% | 100.0% |

Note: NS, negative symptoms; P, paranoia; H, hallucinations.

**Table S3.20. Logistic regression analysis of negative symptoms and (paranoia or hallucinations) with cannabis use by age 16 years^a^**

| Comparison | n | OR (95% CI) | P-value |
| --- | --- | --- | --- |
| NS only vs neither | 5205 | 1.100 (0.813 to 1.490) | 0.536 |
| (P or H) only vs neither | 5736 | 1.896 (1.543 to 2.331) | <0.001 |
| NS+(P or H) vs neither | 4878 | 3.103 (2.285 to 4.215) | <0.001 |

Note: ^a^Generalized estimating equations (GEE) approach, adjusted for birth order, sex, age ~16 years when returned psychotic-like experience questionnaires, and socioeconomic status at 1st contact. OR, odds ratio; NS, negative symptoms; P, paranoia; H, hallucinations.

**Table S3.21. Post hoc analysis - logistic regression analysis of negative symptoms and (paranoia or hallucinations) with cannabis use by age 16 years^a^**

| Comparison | n | OR (95% CI) | P-value |
| --- | --- | --- | --- |
| NS+(P or H) vs (P or H) only | 1626 | 1.676 (1.218 to 2.306) | 0.002 |

Note: ^a^Generalized estimating equations (GEE) approach, adjusted for birth order, sex, age ~16 years when returned psychotic-like experience questionnaires, and socioeconomic status at 1st contact. OR, odds ratio; NS, negative symptoms; P, paranoia; H, hallucinations.

**Life satisfaction age 16 years**

| **Table S3.22. Descriptive statistics of negative symptoms and (paranoia or hallucinations) with life satisfaction age 16 years** | | | | | | |
| --- | --- | --- | --- | --- | --- | --- |
| Life satisfaction score (transformed: reverse score then log_10_ then reverse again) | | | | | | |
| NS and (P or H)  top 15% | N | Mean | Std. Deviation | Median | Minimum | Maximum |
| None of NS, P or H | 6139 | 1.5839 | .17143 | 1.5868 | 1.00 | 1.85 |
| NS & (not P nor H) | 1012 | 1.4966 | .18202 | 1.4820 | 1.03 | 1.85 |
| (P or H) & not NS | 1840 | 1.4318 | .17635 | 1.4240 | 1.02 | 1.85 |
| NS & (P or H) | 567 | 1.3479 | .17271 | 1.3271 | 1.00 | 1.85 |
| Total | 9558 | 1.5314 | .18958 | 1.5490 | 1.00 | 1.85 |

Note: Neg sympts, negative symptoms; P, paranoia; H, hallucinations.

**Table S3.23. Linear regression analysis of negative symptoms and (paranoia or hallucinations) with life satisfaction age 16 years^a^**

| Comparison | β (95% CI) | P-value |
| --- | --- | --- |
| NS only vs neither | -0.089 (-0.102 to -0.075) | <0.001 |
| (P or H) only vs neither | -0.150 (-0.160 to -0.140) | <0.001 |
| NS+(P or H) vs neither | -0.233 (-0.249 to -0.217) | <0.001 |

Note: ^a^Generalized estimating equations (GEE) approach, adjusted for birth order, sex, age ~16 years when returned psychotic-like experience questionnaires, and socioeconomic status at 1st contact (n=9057). NS, negative symptoms; P, paranoia; H, hallucinations.

**Table S3.24. Post hoc analysis - linear regression analysis of negative symptoms and (paranoia or hallucinations) with life satisfaction age 16 years^a^**

| Comparison | n | β (95% CI) | P-value |
| --- | --- | --- | --- |
| NS+(P or H) vs (P or H) only | 2268 | -0.082 (-0.100 to -0.065) | <0.001 |

Note: ^a^Generalized estimating equations (GEE) approach, adjusted for birth order, sex, age ~16 years when returned psychotic-like experience questionnaires, and socioeconomic status at 1st contact. NS, negative symptoms; P, paranoia; H, hallucinations.

**GCSE exams total point score age 16 years**

| **Table S3.25. Descriptive statistics of negative symptoms and (paranoia or hallucinations) with GCSE exams total point score age 16 years** | | | | | | |
| --- | --- | --- | --- | --- | --- | --- |
| GCSE exams total point score | | | | | | |
| NS and (P or H)  top 15% | N | Mean | Std. Deviation | Median | Minimum | Maximum |
| None of NS, P or H | 5474 | 88.4507 | 24.18914 | 91.5000 | .00 | 180.50 |
| NS & (not P nor H) | 829 | 74.9475 | 26.91824 | 78.5000 | .00 | 146.00 |
| (P or H) & not NS | 1644 | 86.5517 | 24.28509 | 89.0000 | .00 | 149.00 |
| NS & (P or H) | 464 | 74.0830 | 25.46726 | 77.0000 | .00 | 136.00 |
| Total | 8411 | 85.9560 | 25.03765 | 89.0000 | .00 | 180.50 |

Note: GCSE, General Certificate of Secondary Education; NS, negative symptoms; P, paranoia; H, hallucinations.

**Table S3.26. Linear regression analysis of negative symptoms and (paranoia or hallucinations) with GCSE exams total point score age 16 years^a^**

| Comparison | β (95% CI) | P-value |
| --- | --- | --- |
| NS only vs neither | -10.447 (-12.470 to -8.424) | <0.001 |
| (P or H) only vs neither | -1.369 (-2.676 to -0.062) | 0.040 |
| NS+(P or H) vs neither | -10.156 (-12.548 to -7.763) | <0.001 |

Note: ^a^Generalized estimating equations (GEE) approach, adjusted for birth order, sex, age ~16 years when returned psychotic-like experience questionnaires, and socioeconomic status at 1st contact (n=8012). GCSE, General Certificate of Secondary Education; NS, negative symptoms; P, paranoia; H, hallucinations.

**Twin modelling**

**Table S3.27. Probandwise concordances**

| PLE group | MZ concordance (%) | SS DZ concordance (%) |
| --- | --- | --- |
| NS only | 192/335 (57.3%) | 130/330 (39.4%) |
| (P or H) only | 272/637 (42.7%) | 192/569 (33.7%) |
| NS+(P or H) | 94/208 (45.2%) | 50/165 (30.3%) |

Note: PLE, psychotic-like experiences; MZ, monozygotic; SS DZ, same-sex dizygotic; NS, negative symptoms; P, paranoia; H, hallucinations.

**Table S3.28. Tetrachoric correlations^a^**

| PLE group | MZ (95% CI) | SS DZ (95% CI) |
| --- | --- | --- |
| NS only | 0.82 (0.76 to 0.86) | 0.59 (0.49 to 0.67) |
| (P or H) only | 0.51 (0.43 to 0.58) | 0.34 (0.25 to 0.43) |
| NS+(P or H) | 0.74 (0.65 to 0.81) | 0.59 (0.45 to 0.70) |

Note: ^a^1714 MZ pairs, 1536 SS DZ pairs. Calculated with the same threshold for both twins and both zygosities as this was best-fitting. PLE, psychotic-like experiences; MZ, monozygotic; SS DZ, same-sex dizygotic; NS, negative symptoms; P, paranoia; H, hallucinations.

**Table S3.29. Parameter estimates for the ACE model^a^**

| PLE group | a^2^ (95% CI) | c^2^ (95% CI) | e^2^ (95% CI) |
| --- | --- | --- | --- |
| NS only | 0.46 (0.25 to 0.67) | 0.36 (0.23 to 0.54) | 0.18 (0.14 to 0.24) |
| (P or H) only | 0.33 (0.09 to 0.56) | 0.18 (0.00 to 0.38) | 0.49 (0.42 to 0.57) |
| NS+(P or H) | 0.30 (0.02 to 0.61) | 0.44 (0.16 to 0.68) | 0.26 (0.19 to 0.35) |

Note: ^a^1714 MZ pairs, 1536 SS DZ pairs. ACE model, twin analysis model including additive genetic, common environmental, and individual-specific environmental effects; PLE, psychotic-like experiences; a^2^, c^2^, e^2^, variance in liability due to additive genetic effects (heritability – also symbolised by h^2^), common environmental effects and individual-specific environmental effects; NS, negative symptoms; P, paranoia; H, hallucinations; MZ, monozygotic; SS DZ, same-sex dizygotic.
