# Supplementary material for: Psychotic‐Like Experiences in Adolescence Occurring in Combination or Isolation: Associations with Schizophrenia Risk Factors
Source: Psychiatr Res Clin Pract. 2021 Jan 18;3(2):67–75. doi: 10.1176/appi.prcp.20200010 (PMC8609425; doi:10.1176/appi.prcp.20200010)
Supplement: Supplementary file 5 — Supplementary Material 5 [file RCP2-3-67-s003.doc]

Online supplement for Cardno AG et al., Psychotic-like experiences in adolescence occurring in combination or isolation: associations with schizophrenia risk factors

**SUPPLEMENTARY RESULTS (4): Analysis of the Schizophrenia Polygenic Risk Score**

**Table S4.01. Logistic regression analysis of paranoia and hallucinations with schizophrenia polygenic risk score^a^**

| PLE Group | n | OR (95% CI) | P-value |
| --- | --- | --- | --- |
| P only vs neither | 3272 | 1.150 (0.918 to 1.441) | 0.224 |
| H only vs neither | 3375 | 1.165 (0.956 to 1.421) | 0.131 |
| P+H vs neither | 3216 | 0.908 (0.709 to 1.162) | 0.442 |

Note: ^a^adjusted for first 10 principal components and genotyping array. PLE, psychotic-like experiences; OR, odds ratio; P, paranoia; H, hallucinations.

**Table S4.02. Logistic regression analysis of cognitive disorganisation and (paranoia or hallucinations) with schizophrenia polygenic risk score^a^**

| PLE Group | n | OR (95% CI) | P-value |
| --- | --- | --- | --- |
| CD only vs neither | 2953 | 0.994 (0.767 to 1.288) | 0.964 |
| (P or H) only vs neither | 3411 | 1.095 (0.932 to 1.286) | 0.270 |
| CD+(P or H) vs neither | 3018 | 1.066 (0.844 to 1.345) | 0.592 |

Note: ^a^adjusted for first 10 principal components and genotyping array. PLE, psychotic-like experiences; OR, odds ratio; CD, cognitive disorganisation; P, paranoia; H, hallucinations.

**Table S4.03. Logistic regression analysis of negative symptoms and (paranoia or hallucinations) with schizophrenia polygenic risk score^a^**

| PLE Group | n | OR (95% CI) | P-value |
| --- | --- | --- | --- |
| NS only vs neither | 2937 | 1.041 (0.847 to 1.279) | 0.701 |
| (P or H) only vs neither | 3303 | 1.070 (0.915 to 1.251) | 0.396 |
| NS+(P or H) vs neither | 2766 | 1.114 (0.856 to 1.450) | 0.421 |

Note: ^a^adjusted for first 10 principal components and genotyping array. PLE, psychotic-like experiences; OR, odds ratio; NS, negative symptoms; P, paranoia; H, hallucinations.
