# Supplementary material for: Psychotic‐Like Experiences in Adolescence Occurring in Combination or Isolation: Associations with Schizophrenia Risk Factors
Source: Psychiatr Res Clin Pract. 2021 Jan 18;3(2):67–75. doi: 10.1176/appi.prcp.20200010 (PMC8609425; doi:10.1176/appi.prcp.20200010)
Supplement: Supplementary file 6 — Supplementary Material 6 [file RCP2-3-67-s002.doc]

Online supplement for Cardno AG et al., Psychotic-like experiences in adolescence occurring in combination or isolation: associations with schizophrenia risk factors

**SUPPLEMENTARY RESULTS (5): Secondary Analysis of Cognitive Disorganisation and Negative Symptoms**

| **CONTENTS** | **Page** |
| --- | --- |
| Summary of secondary analysis of cognitive disorganisation and negative symptoms | 2 |
| Demographic variables | 4 |
| Family history of schizophrenia | 5 |
| Paternal age | 6 |
| Ethnicity | 7 |
| Obstetric complications | 8 |
| Vocabulary | 9 |
| General cognitive ability | 10 |
| Bullying victimization | 11 |
| Cannabis use | 12 |
| Life satisfaction | 13 |
| GCSE score | 14 |
| Twin heritability | 15 |
| Supplementary analysis of schizophrenia polygenic risk score | 16 |

| **Table S5.01. Summary of secondary analysis of cognitive disorganisation and negative symptoms** | |
| --- | --- |
| Schizophrenia-relevant variable | PLE groups^a^ |
|  | Cognitive disorganisation (CD) and Negative symptoms (NS) |
| Logistic regression analysis  (effect measure: odds ratio; no effect = 1) |  |
| Family history of schizophrenia | CD+NS** > CD > NS  2.44 1.45 1.44 |
| Paternal age | [CD] > [NS] > [CD+NS]  0.997 0.991 0.987 |
| Ethnicity | NS** > CD > [CD+NS]  1.64 1.10 0.70 |
| Obstetric complications | NS** > CD+NS > CD  2.38 1.57 1.05 |
| Vocabulary | CD** > CD+NS > NS  1.007 1.005 1.004 |
| General cognitive ability | CD+NS*** > NS*** > CD***  1.57 1.32 1.23 |
| Bullying victimization | CD+NS*** >^‡‡^ CD*** > NS***  1.53 1.30 1.11 |
| Cannabis use | CD+NS*** > CD*** > NS*  2.59 2.21 1.40 |
| Linear regression analysis (effect measure: β; no effect = 0) |  |
| Life satisfaction | CD+NS*** >^‡‡‡^ CD*** > NS***  0.23 0.17 0.09 |
| GCSE score | CD+NS*** >^‡‡‡^ NS*** > CD***  16.76 8.97 6.18 |
|  |  |
| Twin heritability  (effect measure: h^2^; no effect = 0, maximum = 1) | CD+NS^🞎^ > NS^🞎^ > CD  0.67 0.63 0.05 |
|  |  |
| Overall test of association with schizophrenia-relevant variables  Proportion of comparisons where the co-occurring PLE group had the strongest association^b^ | Proportion: 7/10 (0.70)  P-value: 0.014 |
| ^a^Effects given to two decimal places, expanding to three decimal places where required to show order of effects, and presented such that higher values indicate stronger association with schizophrenia-relevant variable in the direction found for schizophrenia.  ^b^Proportion of comparisons where the co-occurring PLE group had the nominally strongest association among the comparisons where at least one association was nominally significant; p-value is for one sample binomial test, two-tailed, based on a null proportion of 0.33.  > indicates relative sizes of trends. [ ] indicates trend in opposite direction to that expected. *p<0.05, **p<0.01, ***p<0.001 for association with the particular PLE group versus baseline of neither PLE present. Where the group of co-occurring PLEs (CD+NS) had the nominally strongest association, post hoc ^‡^p<0.05, ^‡‡^p<0.01, ^‡‡‡^p<0.001 is for the association with the co-occurring PLE group versus the individual PLE with the next strongest association.  Generalized estimating equations (GEE) logistic or linear regression analysis adjusted for birth order, sex, age ~16y when PLE questionnaires completed. Vocabulary age 2y also adjusted for age ~2y when vocabulary assessed. Where significant association additional adjustment for socioeconomic status at 1^st^ contact. GCSE=General Certificate of Secondary Education.  Heritability (h^2^) based on ACE model, comprising additive genetic, common environmental and individual-specific environmental effects, in monozygotic and same-sex dizygotic pairs;  ^🞎^ indicates 95% CIs not including zero and treated as p<0.05. | |

**Detailed Results**

| **TABLE S5.02.** **Demographic Variables for Psychotic-Like Experience (PLE) Groups** | | | | | | | |
| --- | --- | --- | --- | --- | --- | --- | --- |
| PLE Group^a^ | Demographic Variable | | | | | | |
|  | No. Individual Twins | | Sex^b^ | Age in Years | | SES at First Contact^c^ | |
|  | N | % | % Male | Mean | SD | Mean | SD |
| Cognitive disorganisation (CD) and negative symptoms (NS)  (N=9584) |  | |  |  | |  | |
| Neither CD nor NS | 7048 | 73.5 | 45.5 | 16.3 | 0.68 | 0.29 | 0.98 |
| CD and not NS | 950 | 9.9 | 28.6 | 16.4 | 0.67 | 0.17 | 0.99 |
| NS and not CD | 1214 | 12.7 | 53.0 | 16.3 | 0.69 | 0.01 | 0.99 |
| Both CD and NS | 372 | 3.9 | 36.6 | 16.3 | 0.68 | -0.16 | 0.98 |
|  |  | |  |  | |  | |
| ^a^PLE was defined as present if scoring in the top ~15% of the sample.  ^b^Sex distribution differed significantly between PLE groups (χ^2^ = 145.01, df 3, p<0.001).  ^c^SES distribution differed significantly between PLE groups (F = 46.15, df 9080, p<0.001).  SES=family socioeconomic status as a standardized composite score based on parental qualifications and employment - higher score means higher socioeconomic status. | | | | | | | |

**Family history of schizophrenia in a 1^st^ or 2^nd^ degree relative**

| **Table S5.03. Cross tabulation of cognitive disorganisation and negative symptoms by family history of schizophrenia** | | | | | |
| --- | --- | --- | --- | --- | --- |
|  | | | Family history of schizophrenia | | Total |
|  |  |  | No | Yes |  |
| CD and NS  top 15% | neither CD nor NS | Count | 6851 | 174 | 7025 |
|  |  | % | 97.5% | 2.5% | 100.0% |
|  | CD and not NS | Count | 913 | 30 | 943 |
|  |  | % | 96.8% | 3.2% | 100.0% |
|  | NS and not CD | Count | 1156 | 42 | 1198 |
|  |  | % | 96.5% | 3.5% | 100.0% |
|  | both CD and NS | Count | 350 | 20 | 370 |
|  |  | % | 94.6% | 5.4% | 100.0% |
| Total | | Count | 9270 | 266 | 9536 |
|  |  | % | 97.2% | 2.8% | 100.0% |

Note: CD, cognitive disorganisation; NS, negative symptoms.

**Table S5.04. Logistic regression analysis of cognitive disorganisation and negative symptoms with family history of schizophrenia^a^**

| Comparison | n | OR (95% CI) | P-value |
| --- | --- | --- | --- |
| CD only vs neither | 7601 | 1.446 (0.920 to 2.274) | 0.110 |
| NS only vs neither | 7807 | 1.439 (0.940 to 2.202) | 0.094 |
| CD+NS vs neither | 7045 | 2.438 (1.368 to 4.345) | 0.003 |

Note: ^a^Generalized estimating equations (GEE) approach, adjusted for birth order, sex, age ~16 years when returned psychotic-like experience questionnaires, and socioeconomic status at 1st contact. OR, odds ratio; CD, cognitive disorganisation; NS, negative symptoms.

**Table S5.05. Post hoc analysis – logistic regression analysis of cognitive disorganisation and negative symptoms with family history of schizophrenia^a^**

| Comparison | n | OR (95% CI) | P-value |
| --- | --- | --- | --- |
| CD+NS vs CD only | 1232 | 1.686 (0.868 to 3.273) | 0.123 |

Note: ^a^Generalized estimating equations (GEE) approach, adjusted for birth order, sex, age ~16 years when returned psychotic-like experience questionnaires, and socioeconomic status at 1st contact. OR, odds ratio; CD, cognitive disorganisation; NS, negative symptoms.

**Paternal age**

| **Table S5.06. Descriptive statistics of cognitive disorganisation and negative symptoms with paternal age** | | | | | | |
| --- | --- | --- | --- | --- | --- | --- |
| Age in years of natural father at time of birth of twins | | | | | | |
| CD and NS  top 15% | N | Mean | Std. Deviation | Median | Minimum | Maximum |
| neither CD nor NS | 6549 | 33.7780 | 5.56308 | 33.1800 | 16.94 | 59.67 |
| CD and not NS | 874 | 33.6566 | 5.53362 | 33.4264 | 16.94 | 61.23 |
| NS and not CD | 1073 | 33.5326 | 5.82760 | 33.0678 | 17.94 | 58.31 |
| both CD and NS | 322 | 33.3941 | 6.13742 | 32.7050 | 18.45 | 61.23 |
| Total | 8818 | 33.7221 | 5.61489 | 33.1732 | 16.94 | 61.23 |

Note: CD, cognitive disorganisation; NS, negative symptoms.

**Table S5.07. Logistic regression analysis of cognitive disorganisation and negative symptoms with paternal age^a^**

| Comparison | n | OR (95% CI) | P-value |
| --- | --- | --- | --- |
| CD only vs neither | 7423 | 0.997 (0.984 to 1.011) | 0.686 |
| NS only vs neither | 7622 | 0.991 (0.977 to 1.006) | 0.238 |
| CD+NS vs neither | 6871 | 0.987 (0.963 to 1.012) | 0.320 |

Note: ^a^Generalized estimating equations (GEE) approach, adjusted for birth order, sex, and age ~16 years when returned psychotic-like experience questionnaires. OR, odds ratio; CD, cognitive disorganisation; NS, negative symptoms.

| **Table S5.08. Cross tabulation of cognitive disorganisation and negative symptoms by paternal age in 10 year bands** | | | | | | | | |
| --- | --- | --- | --- | --- | --- | --- | --- | --- |
|  | | | Age of father in 10 year bands | | | | | Total |
|  |  |  | <25y | 25-34y | 35-44y | 45-54y | 55y+ |  |
|  | neither CD nor NS | Count | 257 | 3808 | 2246 | 226 | 12 | 6549 |
|  |  | % | 3.9% | 58.1% | 34.3% | 3.5% | 0.2% | 100.0% |
|  | CD and not NS | Count | 46 | 498 | 304 | 25 | 1 | 874 |
|  |  | % | 5.3% | 57.0% | 34.8% | 2.9% | 0.1% | 100.0% |
|  | NS and not CD | Count | 60 | 602 | 372 | 36 | 3 | 1073 |
|  |  | % | 5.6% | 56.1% | 34.7% | 3.4% | 0.3% | 100.0% |
|  | both CD and NS | Count | 13 | 199 | 97 | 11 | 2 | 322 |
|  |  | % | 4.0% | 61.8% | 30.1% | 3.4% | 0.6% | 100.0% |
| Total | | Count | 376 | 5107 | 3019 | 298 | 18 | 8818 |
|  |  | % | 4.3% | 57.9% | 34.2% | 3.4% | 0.2% | 100.0% |

Note: y, years; CD, cognitive disorganisation; NS, negative symptoms.

**Ethnic minority status**

| **Table S5.09. Cross tabulation of cognitive disorganisation and negative symptoms by ethnicity** | | | | | |
| --- | --- | --- | --- | --- | --- |
|  | | | Ethnicity of twins (1=White, 0=Other) | | Total |
|  |  |  | 0 | 1 |  |
| CD and NS  top 15% | neither CD nor NS | Count | 426 | 6612 | 7038 |
|  |  | % | 6.1% | 93.9% | 100.0% |
|  | CD and not NS | Count | 63 | 883 | 946 |
|  |  | % | 6.7% | 93.3% | 100.0% |
|  | NS and not CD | Count | 114 | 1098 | 1212 |
|  |  | % | 9.4% | 90.6% | 100.0% |
|  | both CD and NS | Count | 20 | 350 | 370 |
|  |  | % | 5.4% | 94.6% | 100.0% |
| Total | | Count | 623 | 8943 | 9566 |
|  |  | % | 6.5% | 93.5% | 100.0% |

Note: CD, cognitive disorganisation; NS, negative symptoms.

**Table S5.10. Logistic regression analysis of cognitive disorganisation and negative symptoms with ethnicity^a^**

| Comparison | n | OR (95% CI) | P-value |
| --- | --- | --- | --- |
| CD only vs neither | 7619 | 1.100 (0.811 to 1.492) | 0.538 |
| NS only vs neither | 7831 | 1.639 (1.233 to 2.179) | 0.001 |
| CD+NS vs neither | 7061 | 0.704 (0.375 to 1.320) | 0.274 |

Note: ^a^Generalized estimating equations (GEE) approach, adjusted for birth order, sex, age ~16 years when returned psychotic-like experience questionnaires, and socioeconomic status at 1st contact. OR, odds ratio; CD, cognitive disorganisation; NS, negative symptoms.

**Obstetric complications**

| **Table S5.11. Descriptive statistics of cognitive disorganisation and negative symptoms with obstetric complications** | | | | | | |
| --- | --- | --- | --- | --- | --- | --- |
| Obstetric complications score | | | | | | |
| CD and NS  top 15% | N | Mean | Std. Deviation | Median | Minimum | Maximum |
| neither CD nor NS | 6990 | .2073 | .14094 | .1765 | .00 | .92 |
| CD and not NS | 943 | .2050 | .13694 | .1765 | .00 | .82 |
| NS and not CD | 1197 | .2216 | .14291 | .2000 | .00 | .83 |
| both CD and NS | 368 | .2162 | .13425 | .1875 | .00 | .64 |
| Total | 9498 | .2092 | .14062 | .1765 | .00 | .92 |

Note: CD, cognitive disorganisation; NS, negative symptoms.

**Table S5.12. Logistic regression analysis of cognitive disorganisation and negative symptoms with obstetric complications^a^**

| Comparison | n | OR (95% CI) | P-value |
| --- | --- | --- | --- |
| CD only vs neither | 7578 | 1.052 (0.620 to 1.785) | 0.850 |
| NS only vs neither | 7778 | 2.381 (1.456 to 3.892) | 0.001 |
| CD+NS vs neither | 7019 | 1.570 (0.735 to 3.356) | 0.244 |

Note: ^a^Generalized estimating equations (GEE) approach, adjusted for birth order, sex, age ~16 years when returned psychotic-like experience questionnaires, and socioeconomic status at 1st contact. OR, odds ratio; CD, cognitive disorganisation; NS, negative symptoms.

**Vocabulary age 2 years**

| **Table S5.13. Descriptive statistics of cognitive disorganisation and negative symptoms with vocabulary age 2 years** | | | | | | |
| --- | --- | --- | --- | --- | --- | --- |
| Vocabulary total score | | | | | | |
| CD and NS  top 15% | N | Mean | Std. Deviation | Median | Minimum | Maximum |
| neither CD nor NS | 3708 | 49.56 | 24.630 | 48.00 | 1 | 100 |
| CD and not NS | 530 | 47.03 | 24.318 | 46.00 | 2 | 100 |
| NS and not CD | 619 | 46.27 | 25.241 | 45.00 | 0 | 100 |
| both CD and NS | 182 | 45.33 | 24.574 | 43.50 | 0 | 100 |
| Total | 5039 | 48.74 | 24.704 | 47.00 | 0 | 100 |

Note: CD, cognitive disorganisation; NS, negative symptoms.

**Table S5.14. Logistic regression analysis of cognitive disorganisation and negative symptoms with vocabulary age 2 years^a^**

| Comparison | n | OR (95% CI) | P-value |
| --- | --- | --- | --- |
| CD only vs neither | 4069 | 0.993 (0.989 to 0.998) | 0.002 |
| NS only vs neither | 4143 | 0.996 (0.992 to 1.001) | 0.097 |
| CD+NS vs neither | 3732 | 0.995 (0.988 to 1.002) | 0.137 |

Note: ^a^Generalized estimating equations (GEE) approach, adjusted for birth order, sex, age ~16 years when returned psychotic-like experience questionnaires, age ~2 years when vocabulary assessed, and socioeconomic status at 1st contact. OR, odds ratio; CD, cognitive disorganisation; NS, negative symptoms.

**General cognition age 12 years**

| **Table S5.15. Descriptive statistics of cognitive disorganisation and negative symptoms with general cognition age 12 years** | | | | | | |
| --- | --- | --- | --- | --- | --- | --- |
| General cognition standardised score | | | | | | |
| CD and NS  top 15% | N | Mean | Std. Deviation | Median | Minimum | Maximum |
| neither CD nor NS | 4604 | .118050 | .9705513 | .173116 | -3.5134 | 2.8111 |
| CD and not NS | 593 | -.059589 | .9815966 | -.078863 | -3.3285 | 2.2359 |
| NS and not CD | 736 | -.183028 | 1.0505826 | -.139880 | -3.5933 | 2.3563 |
| both CD and NS | 206 | -.429781 | .9907270 | -.348985 | -3.6803 | 1.9146 |
| Total | 6139 | .046412 | .9915330 | .093829 | -3.6803 | 2.8111 |

Note: CD, cognitive disorganisation; NS, negative symptoms.

**Table S5.16. Logistic regression analysis of cognitive disorganisation and negative symptoms with general cognition age 12 years^a^**

| Comparison | n | OR (95% CI) | P-value |
| --- | --- | --- | --- |
| CD only vs neither | 5003 | 0.815 (0.738 to 0.901) | <0.001 |
| NS only vs neither | 5124 | 0.758 (0.687 to 0.836) | <0.001 |
| CD+NS vs neither | 4628 | 0.638 (0.540 to 0.754) | <0.001 |

Note: ^a^Generalized estimating equations (GEE) approach, adjusted for birth order, sex, age ~16 years when returned psychotic-like experience questionnaires, and socioeconomic status at 1st contact. OR, odds ratio; CD, cognitive disorganisation; NS, negative symptoms.

**Table S5.17. Post hoc analysis - logistic regression analysis of cognitive disorganisation and negative symptoms with general cognition age 12 years^a^**

| Comparison | n | OR (95% CI) | P-value |
| --- | --- | --- | --- |
| CD+NS vs NS only | 880 | 0.867 (0.730 to 1.030) | 0.104 |

Note: ^a^Generalized estimating equations (GEE) approach, adjusted for birth order, sex, age ~16 years when returned psychotic-like experience questionnaires, and socioeconomic status at 1st contact. OR, odds ratio; CD, cognitive disorganisation; NS, negative symptoms.

**Bullying victimization age 12 years**

| **Table S5.18. Descriptive statistics of cognitive disorganisation and negative symptoms with bullying victimization age 12 years** | | | | | | |
| --- | --- | --- | --- | --- | --- | --- |
| Victimization total score (square root transformation) | | | | | | |
| CD and NS  top 15% | N | Mean | Std. Deviation | Median | Minimum | Maximum |
| neither CD nor NS | 5685 | 2.2047 | 1.43800 | 2.2361 | .00 | 5.66 |
| CD and not NS | 760 | 2.6633 | 1.44001 | 2.6458 | .00 | 5.66 |
| NS and not CD | 938 | 2.4869 | 1.46110 | 2.6458 | .00 | 5.66 |
| both CD and NS | 281 | 3.0797 | 1.29585 | 3.1623 | .00 | 5.66 |
| Total | 7664 | 2.3168 | 1.45180 | 2.2361 | .00 | 5.66 |

Note: CD, cognitive disorganisation; NS, negative symptoms.

**Table S5.19. Logistic regression analysis of cognitive disorganisation and negative symptoms with bullying victimization age 12 years^a^**

| Comparison | n | OR (95% CI) | P-value |
| --- | --- | --- | --- |
| CD only vs neither | 6183 | 1.299 (1.225 to 1.379) | <0.001 |
| NS only vs neither | 6330 | 1.105 (1.045 to 1.169) | <0.001 |
| CD+NS vs neither | 5716 | 1.529 (1.391 to 1.681) | <0.001 |

Note: ^a^Generalized estimating equations (GEE) approach, adjusted for birth order, sex, age ~16 years when returned psychotic-like experience questionnaires, and socioeconomic status at 1st contact. OR, odds ratio; CD, cognitive disorganisation; NS, negative symptoms.

**Table S5.20. Post hoc analysis - logistic regression analysis of cognitive disorganisation and negative symptoms with bullying victimization age 12 years^a^**

| Comparison | n | OR(CI) | P |
| --- | --- | --- | --- |
| CD+NS vs CD only | 977 | 1.197 (1.075 to 1.334) | 0.001 |

Note: ^a^Generalized estimating equations (GEE) approach, adjusted for birth order, sex, age ~16 years when returned psychotic-like experience questionnaires, and socioeconomic status at 1st contact. OR, odds ratio; CD, cognitive disorganisation; NS, negative symptoms.

**Cannabis use by age 16 years**

| **Table S5.21. Cross tabulation of cognitive disorganisation and negative symptoms by cannabis use** | | | | | |
| --- | --- | --- | --- | --- | --- |
|  | | | Ever tried cannabis by age 16 years (0=no, 1=yes) | | Total |
|  |  |  | 0 | 1 |  |
| CD and NS  top 15% | neither CD nor NS | Count | 4891 | 465 | 5356 |
|  |  | % | 91.3% | 8.7% | 100.0% |
|  | CD and not NS | Count | 568 | 104 | 672 |
|  |  | % | 84.5% | 15.5% | 100.0% |
|  | NS and not CD | Count | 805 | 104 | 909 |
|  |  | % | 88.6% | 11.4% | 100.0% |
|  | both CD and NS | Count | 242 | 49 | 291 |
|  |  | % | 83.2% | 16.8% | 100.0% |
| Total | | Count | 6506 | 722 | 7228 |
|  |  | % | 90.0% | 10.0% | 100.0% |

Note: CD, cognitive disorganisation; NS, negative symptoms.

**Table S5.22. Logistic regression analysis of cognitive disorganisation and negative symptoms with cannabis use by age 16 years^a^**

| Comparison | n | OR (95% CI) | P-value |
| --- | --- | --- | --- |
| CD only vs neither | 5740 | 2.213 (1.730 to 2.831) | <0.001 |
| NS only vs neither | 5927 | 1.398 (1.075 to 1.817) | 0.012 |
| CD+NS vs neither | 5362 | 2.591 (1.801 to 3.725) | <0.001 |

Note: ^a^Generalized estimating equations (GEE) approach, adjusted for birth order, sex, age ~16 years when returned psychotic-like experience questionnaires, and socioeconomic status at 1st contact. OR, odds ratio; CD, cognitive disorganisation; NS, negative symptoms.

**Table S5.23. Post hoc analysis - logistic regression analysis of cognitive disorganisation and negative symptoms with cannabis use by age 16 years^a^**

| Comparison | n | OR (95% CI) | P-value |
| --- | --- | --- | --- |
| CD+NS vs CD only | 906 | 1.154 (0.778 to 1.714) | 0.476 |

Note: ^a^Generalized estimating equations (GEE) approach, adjusted for birth order, sex, age ~16 years when returned psychotic-like experience questionnaires, and socioeconomic status at 1st contact. OR, odds ratio; CD, cognitive disorganisation; NS, negative symptoms.

**Life satisfaction age 16 years**

| **Table S5.24. Descriptive statistics of cognitive disorganisation and negative symptoms with life satisfaction age 16 years** | | | | | | |
| --- | --- | --- | --- | --- | --- | --- |
| Life satisfaction score (transformed: reverse score then log_10_ then reverse again) | | | | | | |
| CD and NS  top 15% | N | Mean | Std. Deviation | Median | Minimum | Maximum |
| neither CD nor NS | 7040 | 1.5692 | .17532 | 1.5868 | 1.03 | 1.85 |
| CD and not NS | 950 | 1.3976 | .17631 | 1.3729 | 1.00 | 1.85 |
| NS and not CD | 1211 | 1.4771 | .18764 | 1.4820 | 1.00 | 1.85 |
| both CD and NS | 372 | 1.3327 | .16441 | 1.3059 | 1.02 | 1.85 |
| Total | 9573 | 1.5313 | .18952 | 1.5490 | 1.00 | 1.85 |

Note: CD, cognitive disorganisation; NS, negative symptoms.

**Table S5.25. Linear regression analysis of cognitive disorganisation and negative symptoms with life satisfaction age 16 years^a^**

| Comparison | β (95% CI) | P-value |
| --- | --- | --- |
| CD only vs neither | -0.170 (-0.182 to -0.157) | <0.001 |
| NS only vs neither | -0.092 (-0.105 to -0.080) | <0.001 |
| CD+NS vs neither | -0.234 (-0.252 to -0.216) | <0.001 |

Note: ^a^Generalized estimating equations (GEE) approach, adjusted for birth order, sex, age ~16 years when returned psychotic-like experience questionnaires, and socioeconomic status at 1st contact (n=9071). CD, cognitive disorganisation; NS, negative symptoms.

**Table S5.26. Post hoc analysis - linear regression analysis of cognitive disorganisation and negative symptoms with life satisfaction age 16 years^a^**

| Comparison | n | β (95% CI) | P-value |
| --- | --- | --- | --- |
| CD+NS vs CD only | 1241 | -0.066 (-0.087 to -0.045) | <0.001 |

Note: ^a^Generalized estimating equations (GEE) approach, adjusted for birth order, sex, age ~16 years when returned psychotic-like experience questionnaires, and socioeconomic status at 1st contact. CD, cognitive disorganisation; NS, negative symptoms.

**GCSE exams total point score age 16 years**

| **Table S5.27. Descriptive statistics of cognitive disorganisation and negative symptoms with GCSE exams total point score age 16 years** | | | | | | |
| --- | --- | --- | --- | --- | --- | --- |
| GCSE exams total point score | | | | | | |
| CD and NS  top 15% | N | Mean | Std. Deviation | Median | Minimum | Maximum |
| neither CD nor NS | 6282 | 88.8278 | 24.01552 | 92.0000 | .00 | 180.50 |
| CD and not NS | 841 | 82.0279 | 24.89594 | 85.5000 | .00 | 144.00 |
| NS and not CD | 986 | 76.7399 | 26.15706 | 79.0000 | .00 | 146.00 |
| both CD and NS | 307 | 67.7199 | 26.23551 | 71.5000 | .00 | 135.00 |
| Total | 8416 | 85.9621 | 25.04506 | 89.0000 | .00 | 180.50 |

Note: GCSE, General Certificate of Secondary Education; CD, cognitive disorganisation; NS, negative symptoms.

**Table S5.28. Linear regression analysis of cognitive disorganisation and negative symptoms with GCSE exams total point score age 16 years^a^**

| Comparison | β (95% CI) | P-value |
| --- | --- | --- |
| CD only vs neither | -6.181 (-7.860 to -4.503) | <0.001 |
| NS only vs neither | -8.971 (-10.779 to -7.162) | <0.001 |
| CD+NS vs neither | -16.759 (-19.895 to -13.623) | <0.001 |

Note: ^a^Generalized estimating equations (GEE) approach, adjusted for birth order, sex, age ~16 years when returned psychotic-like experience questionnaires, and socioeconomic status at 1st contact (n=8017). GCSE, General Certificate of Secondary Education; CD, cognitive disorganisation; NS, negative symptoms.

**Table S5.29. Post hoc analysis - linear regression analysis of cognitive disorganisation and negative symptoms with GCSE exams total point score age 16 years^a^**

| Comparison | n | β (95% CI) | P-value |
| --- | --- | --- | --- |
| CD+NS vs NS only | 1193 | -7.793 (-11.236 to -4.350) | <0.001 |

Note: ^a^Generalized estimating equations (GEE) approach, adjusted for birth order, sex, age ~16 years when returned psychotic-like experience questionnaires, and socioeconomic status at 1st contact. GCSE, General Certificate of Secondary Education; CD, cognitive disorganisation; NS, negative symptoms.

**Twin modelling**

**Table S5.30. Twin p**robandwise concordances

| PLE group | MZ concordance (%) | SS DZ concordance (%) |
| --- | --- | --- |
| CD only | 82 / 332 (24.7%) | 76 / 312 (24.4%) |
| NS only | 278 / 428 (65.0%) | 134 / 362 (37.0%) |
| CD+NS | 38 / 117 (32.5%) | 16 / 130 (12.3%) |

Note: PLE, psychotic-like experiences; MZ, monozygotic; SS DZ, same-sex dizygotic; CD, cognitive disorganisation; NS, negative symptoms.

**Table S5.31. Tetrachoric twin correlations^a^**

| PLE group | MZ (95% CI) | SS DZ (95% CI) |
| --- | --- | --- |
| CD only | 0.37 (0.26 to 0.48) | 0.35 (0.23 to 0.46) |
| NS only | 0.86 (0.81 to 0.89) | 0.54 (0.44 to 0.63) |
| CD+NS | 0.68 (0.54 to 0.79) | 0.28 (0.08 to 0.46) |

Note: ^a^1720 MZ pairs, 1534 SS DZ pairs. Calculated with the same threshold for both twins and both zygosities as this was best-fitting. PLE, psychotic-like experiences; MZ, monozygotic; SS DZ, same-sex dizygotic; CD, cognitive disorganisation; NS, negative symptoms.

**Table S5.32. Parameter estimates for the ACE twin model^a^**

| PLE group | a^2^ (95% CI) | c^2^ (95% CI) | e^2^ (95% CI) |
| --- | --- | --- | --- |
| CD only | 0.05 (0.00 to 0.38) | 0.32 (0.06 to 0.44) | 0.63 (0.52 to 0.72) |
| NS only | 0.63 (0.44 to 0.84) | 0.22 (0.02 to 0.40) | 0.14 (0.11 to 0.19) |
| CD+NS | 0.67 (0.33 to 0.77) | 0.00 (0.00 to 0.28) | 0.33 (0.23 to 0.47) |

Note: ^a^1720 MZ pairs, 1534 SS DZ pairs. ACE model, twin analysis model including additive genetic, common environmental, and individual-specific environmental effects; PLE, psychotic-like experiences; a^2^, c^2^, e^2^, variance in liability due to additive genetic effects (heritability – also symbolised by h^2^), common environmental effects and individual-specific environmental effects, respectively; CD, cognitive disorganisation; NS, negative symptoms; MZ, monozygotic; SS DZ, same-sex dizygotic.

**Supplementary analysis of schizophrenia polygenic risk score**

**Table S5.33. Logistic regression analysis of cognitive disorganisation and negative symptoms with polygenic risk score^a^**

| PLE Group | n | OR (95% CI) | P-value |
| --- | --- | --- | --- |
| CD only vs neither | 3305 | 1.132 (0.923 to 1.389) | 0.234 |
| NS only vs neither | 3392 | 1.148 (0.952 to 1.385) | 0.149 |
| CD+NS vs neither | 3061 | 0.807 (0.585 to 1.112) | 0.190 |

Note: ^a^adjusted for first 10 principal components and genotyping array. PLE, psychotic-like experiences; OR, odds ratio; CD, cognitive disorganisation; NS, negative symptoms.
