# Supplementary material for: Psychotic‐Like Experiences in Adolescence Occurring in Combination or Isolation: Associations with Schizophrenia Risk Factors
Source: Psychiatr Res Clin Pract. 2021 Jan 18;3(2):67–75. doi: 10.1176/appi.prcp.20200010 (PMC8609425; doi:10.1176/appi.prcp.20200010)
Supplement: Supplementary file 7 — Supplementary Material 7 [file RCP2-3-67-s001.doc]

Online supplement for Cardno AG et al., Psychotic-like experiences in adolescence occurring in combination or isolation: associations with schizophrenia risk factors

**SUPPLEMENTARY RESULTS (6): Secondary Analysis of Salient Results at 10% Cut-Off**

Results for 15% cut-off also copied here for comparison.

| **CONTENTS** | **Page** |
| --- | --- |
| Paranoia and Hallucinations |  |
| General cognitive ability | 2 |
| Bullying victimization | 3 |
|  |  |
| Paranoia or Hallucinations, and Cognitive Disorgansiation |  |
| General cognitive ability | 4 |
| Bullying victimization | 5 |
|  |  |
| Paranoia or Hallucinations, and Negative Symptoms |  |
| Obstetric complications | 7 |
| General cognitive ability | 8 |
| Bullying victimization | 9 |
|  |  |
| Cognitive Disorganisation and Negative Symptoms |  |
| General cognitive ability | 11 |
| Bullying victimization | 13 |
| Twin heritability | 14 |

**Paranoia and Hallucinations**

**General cognition age 12 years**

**Table S1.13. Logistic regression analysis of paranoia and hallucinations with general cognition age 12 years^a^ (15% cut-off)**

| Comparison | n | OR (95% CI) | P-value |
| --- | --- | --- | --- |
| P only vs neither | 4948 | 1.053 (0.945 to 1.174) | 0.351 |
| H only vs neither | 5019 | 0.919 (0.835 to 1.013) | 0.089 |
| P+H vs neither | 4833 | 1.021 (0.909 to 1.147) | 0.723 |

Note: ^a^Generalized estimating equations (GEE) approach, adjusted for birth order, sex, age ~16 years when returned psychotic-like experiences questionnaires, and socioeconomic status at 1st contact. OR, odds ratio; P, paranoia; H, hallucinations.

**Table S6.01. Logistic regression analysis of paranoia and hallucinations with general cognition age 12 years^a^ (10% cut-off)**

| Comparison | n | OR (95% CI) | P-value |
| --- | --- | --- | --- |
| P only vs neither | 5307 | 0.951 (0.844 to 1.071) | 0.404 |
| H only vs neither | 5299 | 0.953 (0.853 to 1.065) | 0.394 |
| P+H vs neither | 5126 | 1.008 (0.870 to 1.169) | 0.914 |

Note: ^a^Generalized estimating equations (GEE) approach, adjusted for birth order, sex, age ~16 years when returned psychotic-like experiences questionnaires, and socioeconomic status at 1st contact. OR, odds ratio; P, paranoia; H, hallucinations.

**Bullying victimization age 12 years**

**Table S1.15. Logistic regression analysis of paranoia and hallucinations with bullying victimization age 12 years^a^ (15% cut-off)**

| Comparison | n | OR (95% CI) | P-value |
| --- | --- | --- | --- |
| P only vs neither | 6146 | 1.418 (1.330 to 1.511) | <0.001 |
| H only vs neither | 6225 | 1.236 (1.165 to 1.310) | <0.001 |
| P+H vs neither | 5988 | 1.591 (1.472 to 1.719) | <0.001 |

Note: ^a^Generalized estimating equations (GEE) approach, adjusted for birth order, sex, age ~16 years when returned psychotic-like experiences questionnaires, and socioeconomic status at 1st contact. OR, odds ratio; P, paranoia; H, hallucinations.

**Table S1.16. Post hoc analysis - logistic regression analysis of paranoia and hallucinations with bullying victimization age 12 years^a^ (15% cut-off)**

| Comparison | n | OR (95% CI) | P-value |
| --- | --- | --- | --- |
| P+H vs P only | 1122 | 1.124 (1.029 to 1.228) | 0.010 |

Note: ^a^Generalized estimating equations (GEE) approach, adjusted for birth order, sex, age ~16 years when returned psychotic-like experiences questionnaires, and socioeconomic status at 1st contact. OR, odds ratio; P, paranoia; H, hallucinations.

**Table S6.02. Logistic regression analysis of paranoia and hallucinations with bullying victimization age 12 years^a^ (10% cut-off)**

| Comparison | n | OR (95% CI) | P-value |
| --- | --- | --- | --- |
| P only vs neither | 6594 | 1.459 (1.359 to 1.568) | <0.001 |
| H only vs neither | 6579 | 1.285 (1.196 to 1.381) | <0.001 |
| P+H vs neither | 6366 | 1.760 (1.593 to 1.943) | <0.001 |

Note: ^a^Generalized estimating equations (GEE) approach, adjusted for birth order, sex, age ~16 years when returned psychotic-like experiences questionnaires, and socioeconomic status at 1st contact. OR, odds ratio; P, paranoia; H, hallucinations.

**Table S6.03. Post hoc analysis - logistic regression analysis of paranoia and hallucinations with bullying victimization age 12 years^a^ (10% cut-off)**

| Comparison | n | OR (95% CI) | P-value |
| --- | --- | --- | --- |
| P+H vs P only | 768 | 1.207 (1.074 to 1.358) | 0.002 |

Note: ^a^Generalized estimating equations (GEE) approach, adjusted for birth order, sex, age ~16 years when returned psychotic-like experiences questionnaires, and socioeconomic status at 1st contact. OR, odds ratio; P, paranoia; H, hallucinations.

**Paranoia or Hallucinations, and Cognitive Disorgansiation**

**General cognition age 12 years**

**Table S2.14. Logistic regression analysis of cognitive disorganisation and (paranoia or hallucinations) with general cognition age 12 years^a^ (15% cut-off)**

| Comparison | n | OR (95% CI) | P-value |
| --- | --- | --- | --- |
| CD only vs neither | 4437 | 0.749 (0.657 to 0.854) | <0.001 |
| (P or H) only vs neither | 5141 | 1.022 (0.944 to 1.107) | 0.591 |
| CD+(P or H) vs neither | 4546 | 0.851 (0.762 to 0.949) | 0.004 |

Note: : ^a^Generalized estimating equations (GEE) approach, adjusted for birth order, sex, age ~16 years when returned psychotic-like experience questionnaires, and socioeconomic status at 1st contact. OR, odds ratio; CD, cognitive disorganisation; P, paranoia; H, hallucinations.

**Table S6.04. Logistic regression analysis of cognitive disorganisation and (paranoia or hallucinations) with general cognition age 12 years^a^ (10% cut-off)**

| Comparison | n | OR (95% CI) | P-value |
| --- | --- | --- | --- |
| CD only vs neither | 4902 | 0.764 (0.680 to 0.857) | <0.001 |
| (P or H) only vs neither | 5147 | 0.993 (0.905 to 1.091) | 0.886 |
| CD+(P or H) vs neither | 4835 | 0.850 (0.754 to 0.958) | 0.008 |

Note: : ^a^Generalized estimating equations (GEE) approach, adjusted for birth order, sex, age ~16 years when returned psychotic-like experience questionnaires, and socioeconomic status at 1st contact. OR, odds ratio; CD, cognitive disorganisation; P, paranoia; H, hallucinations.

**Bullying victimization age 12 years**

**Table S2.16. Logistic regression analysis of cognitive disorganisation and (paranoia or hallucinations) with bullying victimization age 12 years^a^ (15% cut-off)**

| Comparison | n | OR (95% CI) | P-value |
| --- | --- | --- | --- |
| CD only vs neither | 5495 | 1.259 (1.167 to 1.359) | <0.001 |
| (P or H) only vs neither | 6354 | 1.347 (1.283 to 1.415) | <0.001 |
| CD+(P or H) vs neither | 5635 | 1.579 (1.473 to 1.693) | <0.001 |

Note: ^a^Generalized estimating equations (GEE) approach, adjusted for birth order, sex, age ~16 years when returned psychotic-like experience questionnaires, and socioeconomic status at 1st contact. OR, odds ratio; CD, cognitive disorganisation; P, paranoia; H, hallucinations.

**Table S2.17. Post hoc analysis - logistic regression analysis of cognitive disorganisation and (paranoia or hallucinations) with bullying victimization age 12 years^a^ (15% cut-off)**

| Comparison | n | OR (95% CI) | P-value |
| --- | --- | --- | --- |
| CD+(P or H) vs (P or H) only | 1837 | 1.162 (1.078 to 1.252) | <0.001 |

Note: ^a^Generalized estimating equations (GEE) approach, adjusted for birth order, sex, age ~16 years when returned psychotic-like experience questionnaires, and socioeconomic status at 1st contact. OR, odds ratio; CD, cognitive disorganisation; P, paranoia; H, hallucinations.

**Table S6.05. Logistic regression analysis of cognitive disorganisation and (paranoia or hallucinations) with bullying victimization age 12 years^a^ (10% cut-off)**

| Comparison | n | OR (95% CI) | P-value |
| --- | --- | --- | --- |
| CD only vs neither | 6084 | 1.233 (1.153 to 1.319) | <0.001 |
| (P or H) only vs neither | 6364 | 1.392 (1.314 to 1.476) | <0.001 |
| CD+(P or H) vs neither | 5986 | 1.651 (1.529 to 1.782) | <0.001 |

Note: ^a^Generalized estimating equations (GEE) approach, adjusted for birth order, sex, age ~16 years when returned psychotic-like experience questionnaires, and socioeconomic status at 1st contact. OR, odds ratio; CD, cognitive disorganisation; P, paranoia; H, hallucinations.

**Table S6.06. Post hoc analysis - logistic regression analysis of cognitive disorganisation and (paranoia or hallucinations) with bullying victimization age 12 years^a^ (10% cut-off)**

| Comparison | n | OR (95% CI) | P-value |
| --- | --- | --- | --- |
| CD+(P or H) vs (P or H) only | 1248 | 1.181 (1.084 to 1.287) | <0.001 |

Note: ^a^Generalized estimating equations (GEE) approach, adjusted for birth order, sex, age ~16 years when returned psychotic-like experience questionnaires, and socioeconomic status at 1st contact. OR, odds ratio; CD, cognitive disorganisation; P, paranoia; H, hallucinations.

**Paranoia or Hallucinations, and Negative Symptoms**

**Obstetric complications**

**Table S3.10. Logistic regression analysis of negative symptoms and (paranoia or hallucinations) with obstetric complications^a^ (15% cut-off)**

| Comparison | n | OR (95% CI) | P-value |
| --- | --- | --- | --- |
| NS only vs neither | 6747 | 2.053 (1.198 to 3.519) | 0.009 |
| (P or H) only vs neither | 7571 | 1.085 (0.717 to 1.642) | 0.700 |
| NS+(P or H) vs neither | 6341 | 2.378 (1.234 to 4.584) | 0.010 |

Note: ^a^Generalized estimating equations (GEE) approach, adjusted for birth order, sex, age ~16 years when returned psychotic-like experience questionnaires, and socioeconomic status at 1st contact. OR, odds ratio; NS, negative symptoms; P, paranoia; H, hallucinations.

**Table S3.11. Post hoc analysis - logistic regression analysis of negative symptoms and (paranoia or hallucinations) with obstetric complications^a^ (15% cut-off)**

| Comparison | n | OR (95% CI) | P-value |
| --- | --- | --- | --- |
| NS+(P or H) vs NS only | 1432 | 1.166 (0.510 to 2.665) | 0.715 |

Note: ^a^Generalized estimating equations (GEE) approach, adjusted for birth order, sex, age ~16 years when returned psychotic-like experience questionnaires, and socioeconomic status at 1st contact. OR, odds ratio; NS, negative symptoms; P, paranoia; H, hallucinations.

**Table S6.07. Logistic regression analysis of negative symptoms and (paranoia or hallucinations) with obstetric complications^a^ (10% cut-off)**

| Comparison | n | OR (95% CI) | P-value |
| --- | --- | --- | --- |
| NS only vs neither | 7461 | 3.039 (1.756 to 5.259) | <0.001 |
| (P or H) only vs neither | 8148 | 1.146 (0.748 to 1.755) | 0.531 |
| NS+(P or H) vs neither | 7102 | 2.867 (1.299 to 6.328) | 0.009 |

Note: ^a^Generalized estimating equations (GEE) approach, adjusted for birth order, sex, age ~16 years when returned psychotic-like experience questionnaires, and socioeconomic status at 1st contact. OR, odds ratio; NS, negative symptoms; P, paranoia; H, hallucinations.

**General cognition age 12 years**

**Table S3.15. Logistic regression analysis of negative symptoms and (paranoia or hallucinations) with general cognition age 12 years^a^ (15% cut-off)**

| Comparison | n | OR (95% CI) | P-value |
| --- | --- | --- | --- |
| NS only vs neither | 4423 | 0.711 (0.639 to 0.791) | <0.001 |
| (P or H) only vs neither | 5002 | 0.988 (0.913 to 1.068) | 0.761 |
| NS+(P or H) vs neither | 4196 | 0.816 (0.713 to 0.934) | 0.003 |

Note: ^a^Generalized estimating equations (GEE) approach, adjusted for birth order, sex, age ~16 years when returned psychotic-like experience questionnaires, and socioeconomic status at 1st contact. OR, odds ratio; NS, negative symptoms; P, paranoia; H, hallucinations.

**Table S6.08. Logistic regression analysis of negative symptoms and (paranoia or hallucinations) with general cognition age 12 years^a^ (10% cut-off)**

| Comparison | n | OR (95% CI) | P-value |
| --- | --- | --- | --- |
| NS only vs neither | 4888 | 0.667 (0.594 to 0.749) | <0.001 |
| (P or H) only vs neither | 5352 | 0.984 (0.907 to 1.068) | 0.704 |
| NS+(P or H) vs neither | 4669 | 0.712 (0.596 to 0.851) | <0.001 |

Note: ^a^Generalized estimating equations (GEE) approach, adjusted for birth order, sex, age ~16 years when returned psychotic-like experience questionnaires, and socioeconomic status at 1st contact. OR, odds ratio; NS, negative symptoms; P, paranoia; H, hallucinations.

**Bullying victimization age 12 years**

**Table S3.17. Logistic regression analysis of negative symptoms and (paranoia or hallucinations) with bullying victimization age 12 years^a^ (15% cut-off)**

| Comparison | n | OR (95% CI) | P-value |
| --- | --- | --- | --- |
| NS only vs neither | 5477 | 1.112 (1.046 to 1.183) | 0.001 |
| (P or H) only vs neither | 6183 | 1.369 (1.305 to 1.436) | <0.001 |
| NS+(P or H) vs neither | 5163 | 1.510 (1.394 to 1.636) | <0.001 |

Note: ^a^Generalized estimating equations (GEE) approach, adjusted for birth order, sex, age ~16 years when returned psychotic-like experience questionnaires, and socioeconomic status at 1st contact. OR, odds ratio; NS, negative symptoms; P, paranoia; H, hallucinations.

**Table S3.18. Post hoc analysis - logistic regression analysis of negative symptoms and (paranoia or hallucinations) with bullying victimization age 12 years^a^ (15% cut-off)**

| Comparison | n | OR (95% CI) | P-value |
| --- | --- | --- | --- |
| NS+(P or H) vs (P or H) only | 1832 | 1.116 (1.026 to 1.214) | 0.011 |

Note: ^a^Generalized estimating equations (GEE) approach, adjusted for birth order, sex, age ~16 years when returned psychotic-like experience questionnaires, and socioeconomic status at 1st contact. OR, odds ratio; NS, negative symptoms; P, paranoia; H, hallucinations.

**Table S6.09. Logistic regression analysis of negative symptoms and (paranoia or hallucinations) with bullying victimization age 12 years^a^ (10% cut-off)**

| Comparison | n | OR (95% CI) | P-value |
| --- | --- | --- | --- |
| NS only vs neither | 6065 | 1.117 (1.045 to 1.195) | 0.001 |
| (P or H) only vs neither | 6660 | 1.421 (1.352 to 1.493) | <0.001 |
| NS+(P or H) vs neither | 5792 | 1.607 (1.429 to 1.808) | <0.001 |

Note: ^a^Generalized estimating equations (GEE) approach, adjusted for birth order, sex, age ~16 years when returned psychotic-like experience questionnaires, and socioeconomic status at 1st contact. OR, odds ratio; NS, negative symptoms; P, paranoia; H, hallucinations.

**Table S6.10. Post hoc analysis - logistic regression analysis of negative symptoms and (paranoia or hallucinations) with bullying victimization age 12 years^a^ (10% cut-off)**

| Comparison | n | OR (95% CI) | P-value |
| --- | --- | --- | --- |
| NS+(P or H) vs (P or H) only | 1244 | 1.145 (1.016 to 1.291) | 0.026 |

Note: ^a^Generalized estimating equations (GEE) approach, adjusted for birth order, sex, age ~16 years when returned psychotic-like experience questionnaires, and socioeconomic status at 1st contact. OR, odds ratio; NS, negative symptoms; P, paranoia; H, hallucinations.

**Cognitive Disorganisation and Negative Symptoms**

**General cognition age 12 years**

**Table S5.16. Logistic regression analysis of cognitive disorganisation and negative symptoms with general cognition age 12 years^a^ (15% cut-off)**

| Comparison | n | OR (95% CI) | P-value |
| --- | --- | --- | --- |
| CD only vs neither | 5003 | 0.815 (0.738 to 0.901) | <0.001 |
| NS only vs neither | 5124 | 0.758 (0.687 to 0.836) | <0.001 |
| CD+NS vs neither | 4628 | 0.638 (0.540 to 0.754) | <0.001 |

Note: ^a^Generalized estimating equations (GEE) approach, adjusted for birth order, sex, age ~16 years when returned psychotic-like experience questionnaires, and socioeconomic status at 1st contact. OR, odds ratio; CD, cognitive disorganisation; NS, negative symptoms.

**Table S5.17. Post hoc analysis - logistic regression analysis of cognitive disorganisation and negative symptoms with general cognition age 12 years^a^ (15% cut-off)**

| Comparison | n | OR (95% CI) | P-value |
| --- | --- | --- | --- |
| CD+NS vs NS only | 880 | 0.867 (0.730 to 1.030) | 0.104 |

Note: ^a^Generalized estimating equations (GEE) approach, adjusted for birth order, sex, age ~16 years when returned psychotic-like experience questionnaires, and socioeconomic status at 1st contact. OR, odds ratio; CD, cognitive disorganisation; NS, negative symptoms.

**Table S6.11. Logistic regression analysis of cognitive disorganisation and negative symptoms with general cognition age 12 years^a^ (10% cut-off)**

| Comparison | n | OR (95% CI) | P-value |
| --- | --- | --- | --- |
| CD only vs neither | 5351 | 0.822 (0.750 to 0.902) | <0.001 |
| NS only vs neither | 5131 | 0.691 (0.617 to 0.774) | <0.001 |
| CD+NS vs neither | 4855 | 0.577 (0.476 to 0.700) | <0.001 |

Note: ^a^Generalized estimating equations (GEE) approach, adjusted for birth order, sex, age ~16 years when returned psychotic-like experience questionnaires, and socioeconomic status at 1st contact. OR, odds ratio; CD, cognitive disorganisation; NS, negative symptoms.

**Table S6.12. Post hoc analysis - logistic regression analysis of cognitive disorganisation and negative symptoms with general cognition age 12 years^a^ (10% cut-off)**

| Comparison | n | OR (95% CI) | P-value |
| --- | --- | --- | --- |
| CD+NS vs NS only | 532 | 0.879 (0.712 to 1.084) | 0.228 |

Note: ^a^Generalized estimating equations (GEE) approach, adjusted for birth order, sex, age ~16 years when returned psychotic-like experience questionnaires, and socioeconomic status at 1st contact. OR, odds ratio; CD, cognitive disorganisation; NS, negative symptoms.

**Bullying victimization age 12 years**

**Table S5.19. Logistic regression analysis of cognitive disorganisation and negative symptoms with bullying victimization age 12 years^a^ (15% cut-off)**

| Comparison | n | OR (95% CI) | P-value |
| --- | --- | --- | --- |
| CD only vs neither | 6183 | 1.299 (1.225 to 1.379) | <0.001 |
| NS only vs neither | 6330 | 1.105 (1.045 to 1.169) | <0.001 |
| CD+NS vs neither | 5716 | 1.529 (1.391 to 1.681) | <0.001 |

Note: ^a^Generalized estimating equations (GEE) approach, adjusted for birth order, sex, age ~16 years when returned psychotic-like experience questionnaires, and socioeconomic status at 1st contact. OR, odds ratio; CD, cognitive disorganisation; NS, negative symptoms.

**Table S5.20. Post hoc analysis - logistic regression analysis of cognitive disorganisation and negative symptoms with bullying victimization age 12 years^a^  (15% cut-off)**

| Comparison | n | OR(CI) | P |
| --- | --- | --- | --- |
| CD+NS vs CD only | 977 | 1.197 (1.075 to 1.334) | 0.001 |

Note: ^a^Generalized estimating equations (GEE) approach, adjusted for birth order, sex, age ~16 years when returned psychotic-like experience questionnaires, and socioeconomic status at 1st contact. OR, odds ratio; CD, cognitive disorganisation; NS, negative symptoms.

**Table S6.13. Logistic regression analysis of cognitive disorganisation and negative symptoms with bullying victimization age 12 years^a^ (10% cut-off)**

| Comparison | n | OR (95% CI) | P-value |
| --- | --- | --- | --- |
| CD only vs neither | 6658 | 1.323 (1.253 to 1.398) | <0.001 |
| NS only vs neither | 6341 | 1.134 (1.061 to 1.212) | <0.001 |
| CD+NS vs neither | 6022 | 1.488 (1.326 to 1.669) | <0.001 |

Note: ^a^Generalized estimating equations (GEE) approach, adjusted for birth order, sex, age ~16 years when returned psychotic-like experience questionnaires, and socioeconomic status at 1st contact. OR, odds ratio; CD, cognitive disorganisation; NS, negative symptoms.

**Table S6.14. Post hoc analysis - logistic regression analysis of cognitive disorganisation and negative symptoms with bullying victimization age 12 years^a^  (10% cut-off)**

| Comparison | n | OR(CI) | P |
| --- | --- | --- | --- |
| CD+NS vs CD only | 966 | 1.142 (1.008 to 1.293) | 0.037 |

Note: ^a^Generalized estimating equations (GEE) approach, adjusted for birth order, sex, age ~16 years when returned psychotic-like experience questionnaires, and socioeconomic status at 1st contact. OR, odds ratio; CD, cognitive disorganisation; NS, negative symptoms.

**Heritability estimates and other twin modelling results**

**Table S5.32. Parameter estimates for the ACE twin model^a^ (15% cut-off)**

| PLE group | a^2^ (95% CI) | c^2^ (95% CI) | e^2^ (95% CI) |
| --- | --- | --- | --- |
| CD only | 0.05 (0.00 to 0.38) | 0.32 (0.06 to 0.44) | 0.63 (0.52 to 0.72) |
| NS only | 0.63 (0.44 to 0.84) | 0.22 (0.02 to 0.40) | 0.14 (0.11 to 0.19) |
| CD+NS | 0.67 (0.33 to 0.77) | 0.00 (0.00 to 0.28) | 0.33 (0.23 to 0.47) |

Note: ^a^1720 MZ pairs, 1534 SS DZ pairs. ACE model, twin analysis model including additive genetic, common environmental, and individual-specific environmental effects; PLE, psychotic-like experiences; a^2^, c^2^, e^2^, variance in liability due to additive genetic effects (heritability – also symbolised by h^2^), common environmental effects and individual-specific environmental effects, respectively; CD, cognitive disorganisation; NS, negative symptoms; MZ, monozygotic; SS DZ, same-sex dizygotic.

**Table S6.15. Parameter estimates for the ACE twin model^a^ (10% cut-off)**

| PLE group | a^2^ (95% CI) | c^2^ (95% CI) | e^2^ (95% CI) |
| --- | --- | --- | --- |
| CD only | 0.20 (0.00 to 0.49) | 0.21 (0.00 to 0.42) | 0.59 (0.49 to 0.93) |
| NS only | 0.69 (0.45 to 0.91) | 0.19 (0.00 to 0.41) | 0.13 (0.09 to 0.18) |
| CD+NS | 0.70 (0.41 to 0.82) | 0.00 (0.00 to 0.24) | 0.30 (0.18 to 0.46) |

Note: ^a^1720 MZ pairs, 1534 SS DZ pairs. ACE model, twin analysis model including additive genetic, common environmental, and individual-specific environmental effects; PLE, psychotic-like experiences; a^2^, c^2^, e^2^, variance in liability due to additive genetic effects (heritability – also symbolised by h^2^), common environmental effects and individual-specific environmental effects, respectively; CD, cognitive disorganisation; NS, negative symptoms; MZ, monozygotic; SS DZ, same-sex dizygotic.
